# Toward Evaluation of the Subjective Experience of a General Class of User-Controlled, Robot-Mediated Rehabilitation Technologies for Children with Neuromotor Disability

**DOI:** 10.3390/informatics7040045

**Published:** 2020

**Authors:** Manon Maitland Schladen, Kevin Cleary, Yiannis Koumpouros, Reza Monfaredi, Tyler Salvador, Hadi Fooladi Talari, Jacob Slagle, Catherine Coley, Staci Kovelman, Justine Belschner, Sarah Helen Evans

**Affiliations:** 1 MedStar Health Research Institute, Hyattsville, MD 20782, USA; 2 Department of Rehabilitation Medicine, Georgetown University Medical Center,Washington, DC 20057, USA; 3 Children’s National Medical Center,Washington, DC 20010, USA; 4 Department of Public and Community Health, University ofWest Attica, 12243 Aigaleo, Greece; 5 Children’s Hospital of Philadelphia, Philadelphia, PA 19104, USA

**Keywords:** game-based therapy, robot-mediated therapy, neuromotor disability, cerebral palsy, subjective assessment, patient-centered assessment, caregiver burden, ankle range of motion, ankle strengthening, home exercise program

## Abstract

Technological advances in game-mediated robotics provide an opportunity to engage children with cerebral palsy (CP) and other neuromotor disabilities in more frequent and intensive therapy by making personalized, programmed interventions available 24/7 in children’s homes. Though shown to be clinically effective and feasible to produce, little is known of the subjective factors impacting acceptance of what we term assistive/rehabilitative (A/R) gamebots by their target populations. This research describes the conceptualization phase of an effort to develop a valid and reliable instrument to guide the design of A/R gamebots. We conducted in-depth interviews with 8 children with CP and their families who had trialed an exemplar A/R gamebot, PedBotHome, for 28 days in their homes. The goal was to understand how existing theories and instruments were either appropriate or inappropriate for measuring the subjective experience of A/R gamebots. Key findings were the importance of differentiating the use case of therapy from that of assistance in rehabilitative technology assessment, the need to incorporate the differing perspectives of children with CP and those of their parents into A/R gamebot evaluation, and the potential conflict between the goals of preserving the quality of the experience of game play for the child while also optimizing the intensity and duration of therapy provided during play.

## 1. Introduction

The global incidence and prevalence of brain injuries occurring in the time around birth that ultimately manifest as permanent motor disabilities in children is unknown. The worldwide prevalence of the most commonly diagnosed neuromotor disorder affecting children, cerebral palsy (CP), holds steady at 2.11 per 1000 births [1]. CP is a permanent, movement disorder stemming from nonprogressive disturbances to the brain during gestation or from injury in the postnatal period up to age five [2]. Around 75% of children with CP are ambulatory, but many of these children are literally unable to keep up with their peers as they cannot walk as quickly and fluidly and are at much greater risk for falling [3]. Current gait-therapeutic options for children with CP are insufficient to provide consistent and adequate stretching and strengthening of the muscles that cause walking disorders [4,5]. Targeting key muscles in the ankle is critical to gait remediation [6,7,8,9], but treatment best practice often requires extensive bracing or complex constraint mechanisms [10]. As a result, intervening to stretch and strengthen key muscle groups currently requires time-intensive, in-clinic therapies as well as an intensive home exercise regimen to provide an optimal program for improving gait [5,11,12]. Compliance is difficult to maintain, and children often do not receive the level of care needed [13,14].

The challenge optimal intervention presents, both in terms of clinic scheduling and travel time on the part of the child and family/caregiver, could be lessened through leverage of a home-based robotic platform that delivers stretching and strengthening exercises in proper form, at frequency and intensity most appropriate to the child with CP. The positive effect [15,16] of robot-mediated ankle maneuvers on gait therapeutic targets has been demonstrated, as has the feasibility of the transfer of the technology from lab to clinic [17] and home [18,19,20]. Delivering physical therapy interventions to children with CP in a game-play context (i.e., games used seriously) has likewise been shown to spur interest and motivate engagement fairly consistently across a wide range of therapeutic targets involving both lower and upper extremities [21,22].

Bringing these two strands of research together, robot-guided therapeutic maneuvers and games used seriously, has effectively created a new class of rehabilitative technology, which we will call assistive and/or rehabilitative (A/R) gamebots for ease of reference. The medical device payment structure in the US, for example, as yet has no classification under which A/R gamebots might be reimbursed in a child’s therapy program [23]. The novelty of A/R gamebots evolves from the indirection that is at the heart of their function. First, the appropriate therapeutic regimen is prescribed by the clinician but carried out by the system robot. Second, the child’s attention is redirected from the goal of therapeutic exercise to achieve clinical outcomes to the goal of executing movements to navigate within a game scenario.

Positive evidence supporting the effectiveness of robotic and game-based strategies (separately and in combination), in promoting progress toward motor-therapeutic milestones in CP has been aggregated across several reviews [1,21,22]. However, no work has addressed the emerging drivers of adoption of A/R gamebots or the range of subjective factors underpinning their use either clinically, or in support of the intensive home exercise regimen recommended to effect lasting motor improvement in children with CP [24]. Similarly, there are no valid and reliable tools to guide the design iteration of these technologies, advise trade studies, identify best practice heuristics, or support other decision-making processes inherent in prototype development [25]. Understanding the patient, family, and therapist perspectives is fundamental to measuring the impact of system features and optimizing design and utility.

A systematic review of practices surrounding measurement of users’ subjective experience of generic robotic, assistive, and/or rehabilitative technologies (A/RT), revealed that custom, home-grown instruments, not supported by reliability and validity studies, predominated [26]. Assistive and rehabilitative technologies are often treated synonymously, given that they both focus on the needs of people who experience disability and that their functions can overlap. Assistive technologies (AT) embrace any “item, piece of equipment, software program, or product system that is used to increase, maintain, or improve the functional capabilities of persons with disabilities.” [27]. Rehabilitation technologies (RT), on the other hand, always carry an explicitly therapeutic purpose. They are a more recent technological development and have been influenced by motor learning principles, particularly as enablers of massed practice [28].

Valid and reliable instruments (such as the ATDPA [29] and QUEST 2.0 [30]) designed to assess the subjective experience of (explicitly) assistive technologies (AT) have long been available. Their development pre-dates the increasingly ubiquitous leverage of robotics, with their inherent complexity, in technologies targeting people with disabilities. The PYTHEIA (The word “PYTHEIA” comes from the ancient Greek verb πυνθάνομαι”, which means to be informed. Moreover, Pytheia was the priestess of the Greek god Apollo at the Oracle of Delphi, who, in ecstasy, conveyed the ordination of the god to the person concerned in a way that was usually laconic, difficult and enigmatic), on the other hand, is a valid and reliable instrument designed for evaluation of technologies (assistive or rehabilitative) that incorporate robotics [31]. This newer instrument, while its focus is on the new variables robotics introduce into technology acceptance, carries forward many of the constructs measured in those earlier, widely-used AT instruments. The PYTHEIA further incorporates an innovation of allowing the scale administrator to flexibly replicate item sets to evaluate multiple, individual component functionalities within a given system. In work focused on subjective assessment of dynamic hand exoskeleton orthoses supporting practice of therapeutic tasks at home after stroke, our group identified multiplicity of functionality as one of the key characteristics distinguishing robotic rehabilitation systems from earlier passive mechanical ones [32].

### 1.1. Objective

The objective of this current study is to develop a conceptual framework supporting evaluation of, and decision making around, A/R gamebots. This framework will serve as the foundation for the development and validation of a research and development instrument to assess the subjective experience of users of this generalized class of game/robot rehabilitation technology. Since the generalized must take root in some specific, we grounded our inquiry in the specific task of improving gait in children with neuromotor impairments (as exemplified by CP) through increased opportunity for therapeutic engagement in the home. Lower extremity rehabilitation, specifically, the ankle, as well as the pediatric and home-based applications, further bounded the context of inquiry.

### 1.2. Research Question (RQ1)

How well do

- Existing theories of pediatric home exercise program adherence;
- Published criteria for gauging acceptance of game-mediated therapy in children; and
- A valid and reliable instrument for measuring subjective assessment of specifically robotic assistive and/or rehabilitative technologies (A/RT)

support evaluation of user-controlled, robot-assisted pediatric rehabilitation technology?

## 2. Materials and Methods

### 2.1. Procedure

**Figure 1.**
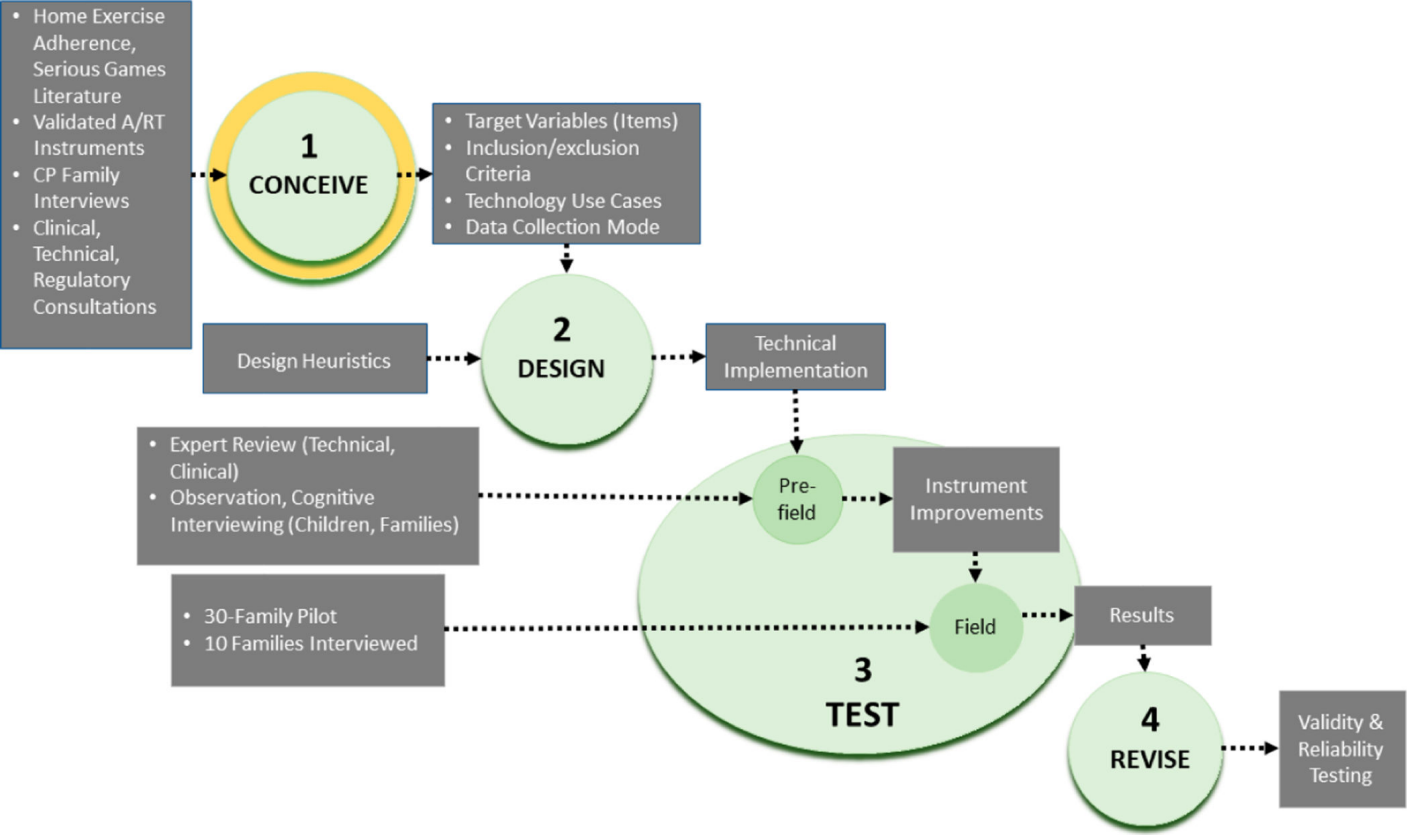
Conceptualization phase (highlighted) of A/R gamebot subjective evaluation in the context of overall instrument development (European Statistical System Model [33]).

To address our research question, we adapted the systematic procedures developed Brancato and colleagues [33] for the European Statistical System (ESS, https://ec.europa.eu/eurostat/web/ess) to guide questionnaire development and testing. See Figure 1. Conceptualization, the focus of this report, is the first component of a recommended 4-phase process: conceive, design, test, and revise. The ESS methods are similar to those used more generally in instrument design in the health sciences [34] and brings the added advantage of a succinct, manualized format. The purpose of the conceptualization phase is to generate candidate assessment items to be refined across the subsequent three phases of the instrument development process. An instrument emerges from the end of this process ready for validity and reliability testing. The sole existing scale measuring the subjective experience of explicitly robotic A/RT, the PYTHEIA, was itself developed according to the ESS process [33]. Table 1 provides a logic model for the A/R gamebot conceptualization phase.

We searched the literature to identify existing studies of adherence in home exercise programs (HEP) for children with CP: those implemented manually (by children and parents) [13,14] and those facilitated by technologies that incorporated games to incentivize engagement [35,36,37,38,39]. We used these inputs in conjunction with items from the PYTHEIA [31] to develop a schedule (see Supplementary Materials) to probe the user experience of seven families who had just completed a month-long, home pilot of PedBotHome, [16,18,19,24] an A/R gamebot prototype designed to promote ankle stretching and strengthening therapeutic exercise.

**Figure 2.**
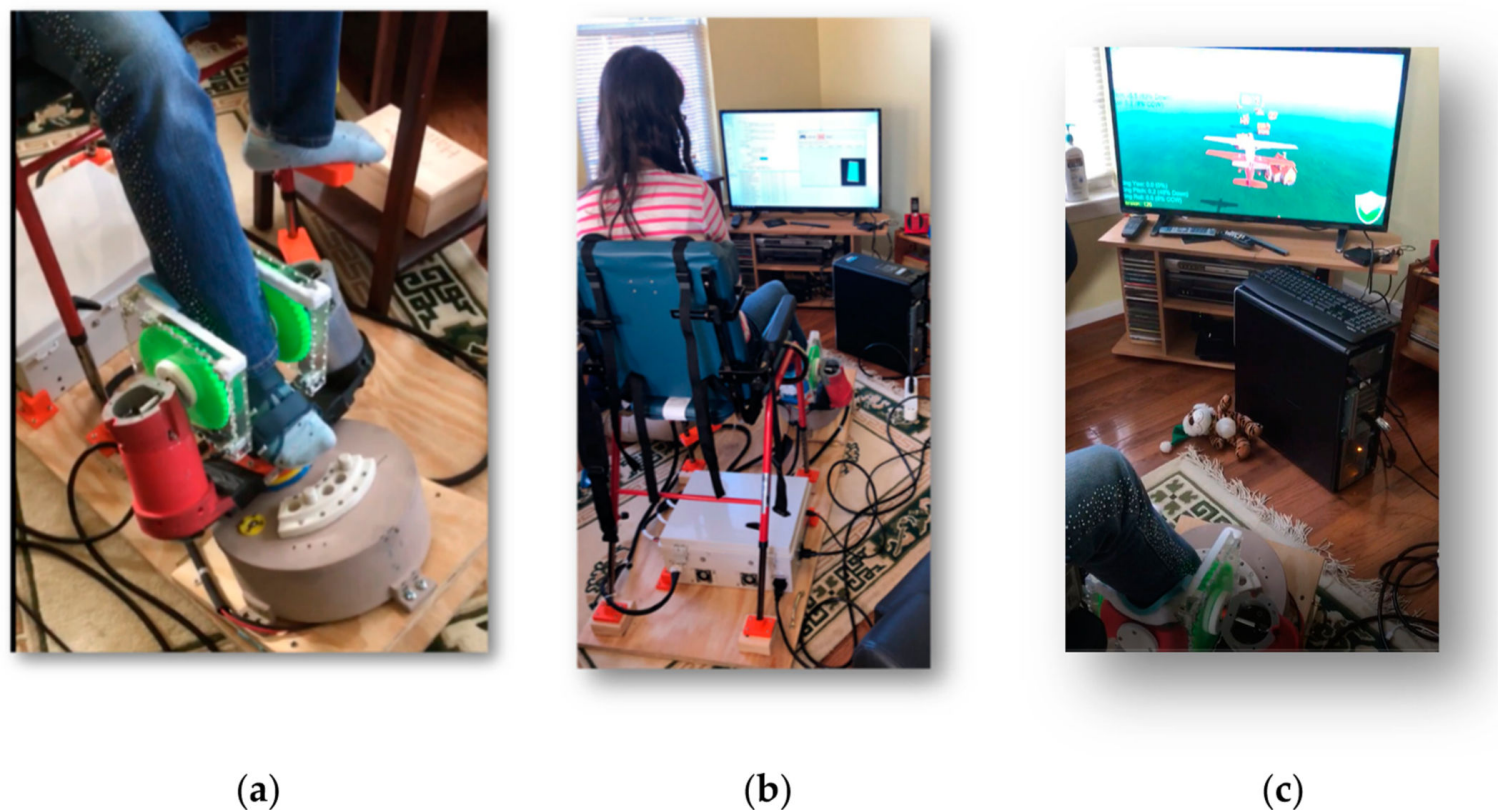
PedBotHome prototype in pilot use in a participant’s home. (**a**) Child’s foot in the robot-assisted controller; (**b**) PedBotHome prototype footprint; (**c**) Flying game interface.

The PedBotHome robotic foot plate provides three degrees of freedom (pitch, yaw, roll). The foot plate is connected to a differential drive mechanism through custom gears printed on a MakerBot Replicator + (http://makerbot.com). Figure 2a shows the custom gears (green). The red cylinder at the lower left is the motor housing. The foot plate functions as a video game controller to engage the child in ankle flexibility and strengthening maneuvers either through free movement (as the child is able to provide) or in assist mode to help the child reach game targets or in resist mode to increase challenge and therapeutic dosage. The angles of rotation are measured using a cell phone (not visible) secured under the bottom of the foot plate.

Figure 2b shows the setup of PedBotHome in a participant’s family room. The screen shows an icon of the cell phone during the setup and calibration phase of PedBotHome The principal game children were asked to interact with daily involved executing various ankle maneuvers—adduction/abduction (yaw), dorsiflexion/plantar flexion (pitch), and inversion/eversion (roll) to fly an airplane through hoops under increasing challenge (see Figure 2c). This airplane game was developed using the Unity (http://unity.com) gaming engine. The therapist accessed the child’s use data remotely and adjusted the level of assistance or resistance to promote therapeutic goals. See our technical paper [19] for a detailed discussion of hardware and software components of PedBotHome.

### 2.2. Participants and Setting

Eight children with CP from seven families living in the Washington, DC, metropolitan area participated in a 28-day pilot of the PedBotHome A/R gamebot. See Table 2. The goal was for each child to carry out a progressive, custom, therapist-prescribed, program of ankle strengthening and range-of-motion exercises at home daily for up to an hour. All participating children were diagnosed at Level I or Level II on the Gross Motor Function Classification System (GMFCS) for CP where Level I represents children who are most able and Level V represents those who are least able with respect to motor function [40]. Children were 9–16 years of age and all but one was female. Children actually used PedBotHome from between 5 and 28 days (18–100% utilization) while it was in their homes. The two very low use records correspond to a family in the process of moving (#5) and difficult-to-resolve technical difficulties (#8).

Interviews with children and their parents (5 mothers, 2 fathers) lasted from one to two hours and took place after the completion of families’ trial of PedBotHome. Six interviews were conducted in-person, face-to-face, in participants’ homes. One of these interviews involved two children with CP who were siblings. One interview was conducted via three-way phone conference: researcher, parent, and child with CP. Two study engineers and one study therapist were present during parts of five of the six, in-person interviews. All spoken interactions were audio-recorded. For the face-to-face interviews, child and parent interaction with the robot was observed and recorded (still photos and video). We subsequently recruited and consulted 24 experts from clinical, regulatory, engineering, and commercial product domains to help us place families’ experience with PedBotHome in the context of access to therapy, device availability, and reimbursement. Audio materials were transcribed; transcripts, family observation notes, as well as notes from consultations with the 24 domain experts were thematically analyzed using NVivo12 (http://qsrinternational.com) computer-assisted qualitative data analysis software (CAQDAS). This research was approved and supervised by IRBear, the institutional review board for Children’s National Medical Center in Washington, DC, USA.

## 3. Results

Results are organized in three parts. First, we present the alignment of families’ experience of the PedBotHome A/R gamebot with factors synthesized from two HEP adherence theories proposed based on work with children with CP. This section (Section 3.1) addresses the first part (RQ1a) of the overarching research question that explores the fit of A/R gamebots within existing pediatric home exercise adherence theory. Second, we present the alignment of the PedBotHome experience with published exergame engagement factors, similarly developed through studies of children with CP. This section (Section 3.2) addresses the RQ1b. Incorporated into the report of each of these alignments results, we make note of whether the PYTHEIA contains a related measure. Finally, we present the direct alignment of PedBotHome experience with the PYTHEIA scale based on parent and child appraisal. This final section (Section 3.3) addresses RQ1c.

### 3.1. Alignment of PedBotHome User Experience with HEP Adherence Theory in CP (RQ1a)

**Figure 3.**
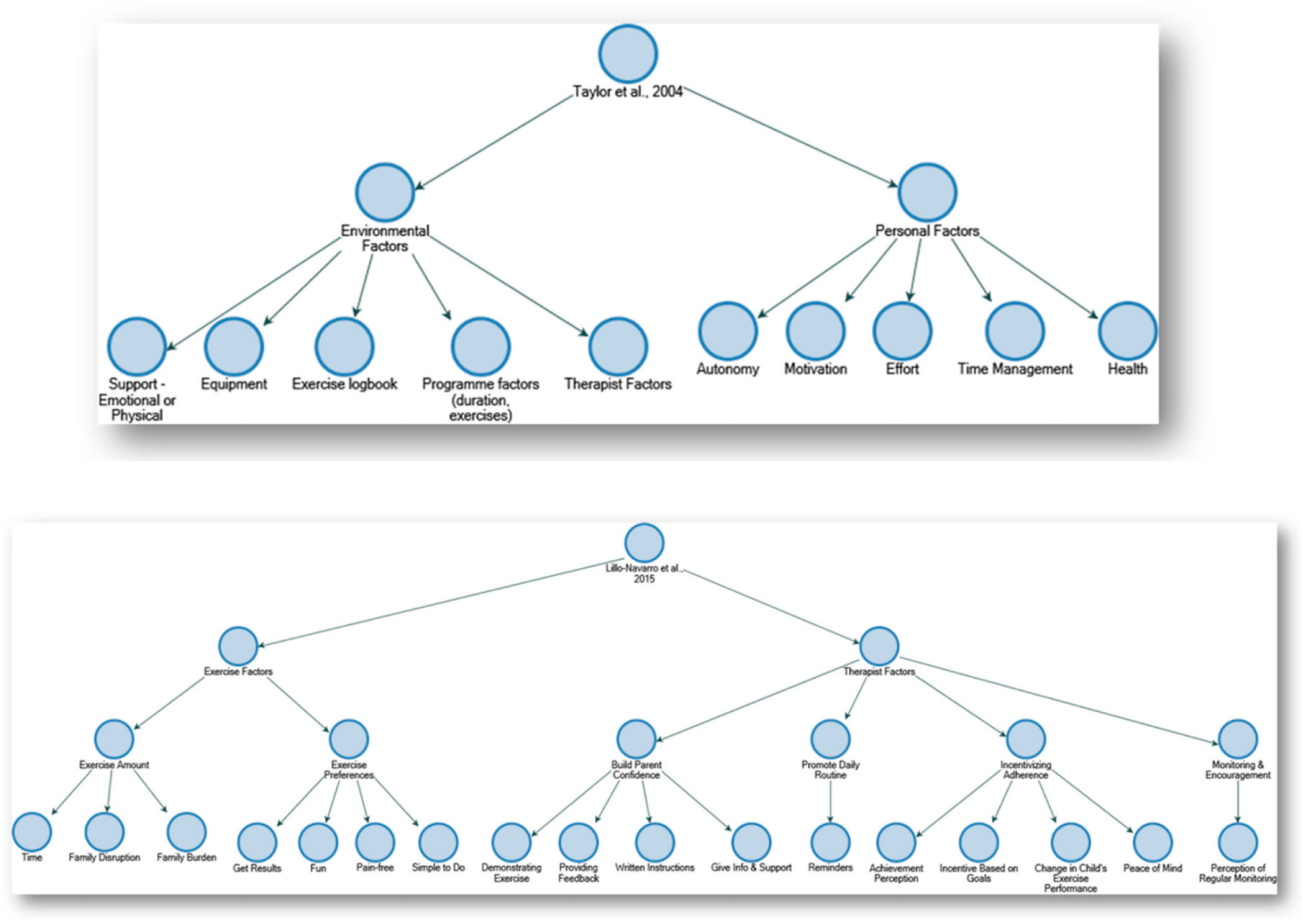
Comparative home exercise program adherence theories.

Theories of adherence to HEP for children with CP have been explicitly proposed by two studies: Taylor et al., 2004 [13] and Lillo-Navarro et al., 2015 [14]. Both of these theories emerged from conventional (i.e., not robotic and not game-mediated) HEP. Taylor and colleagues categorized adherence factors into broad environmental and personal categories. Lillo-Navarro and colleagues focused on the environmental; factors they identified aligned with and effectively expanded upon those proposed by Taylor et al. Figure 3 provides a graphical representation of the factors reported by both studies. A synthesis of these two theories yielded 21 factors that mapped onto three categories of experience: Fit of Exercise Program in the Home Environment, Therapist Support, and Personal Factors. Families’ experience of PedBotHome aligned with 13 factors, did not align (with qualification) with seven factors, and aligned ambiguously with the remaining single factor. See Table 3. We discuss each of these factors below in the context of families’ experience with PedBotHome, noting as well areas of alignment, non-alignment, and ambiguity with the constructs measured by the PYTHEIA.

#### 3.1.1. Fit of Exercise Program in the Home Environment

##### Exercise Equipment

Aligned. PedBotHome provided both the program and the equipment for children’s HEP. Adherence to use of the A/R gamebot was synonymous with adherence to the program. A focus on the centrality of equipment also aligns with the focus of the PYTHEIA: technology evaluation.

##### What the Exercise Is

Aligned. Exercise mediated by the PedBotHome robotic footplate controller consisted of three ankle exercise maneuvers as previous described with neutral, positive (assist), or negative (resist) force added as prescribed by the study therapist. The resist and assist functions created difficulties for children the carrying out the ankle exercises in the PedBotHome prototype.

Girl (age 9): Sometimes the resistance, and assistance for that matter, would go a little crazy.

Her Mom: It wouldn’t let go?

Girl: Sometimes it stopped completely or sometimes while I was up, it would freeze almost—not the—the screen would keep going, but I wouldn’t be able to move my foot. It felt like this invisible wall.

Assessment of functions such as assist/resist aligns with the PYTHEIA’s individual functionalities (IF) item set. See Table 4.

##### Perceived Effectiveness of Exercise

Aligned. Families’ perception of improvements in flexibility and strength contributed to positive appraisal of PedBotHome.

Mom of 13-year-old Girl: [PedBotHome has led to a] big improvement for her! She is able to do things that she could never be able to do before. She’s never been able to do that, [stand] on her tip toe.

Perceived effectiveness aligns with item 2 of the PYTHEIA measuring perceived improvement the target A/RT effects in the individual’s everyday life.

##### Comfort during Exercise

Aligned. Issues of comfort caused children to make adjustments but did not interfere with their completion of daily prescribed exercises using PedBotHome.

Girl (age 11): I started to get this … on the foot piece, on the box … like where you put your foot in, and then there’s the box around it, the clear box around it. There was a screw somewhere in it and it was sticking out a little bit, and it was rubbing against my foot and it hurt. I put a piece of foam on it. It wasn’t permanent. I didn’t glue it on or anything. I just would stuff it there. When I got into the chair, it [the protruding screw] immediately started hurting, even before I started to play the games and stuff. [However] that didn’t discourage me to not do it.

Alignment of physical comfort with the items measured by the PYTHEIA is unclear. Items 2 (improvement to everyday life) and 10 (feeling protected) have some commonality. Items 13–15 deal with specifically social (versus physical) comfort.

##### Perceived Complexity of Doing Exercise

Aligned. Complexity was largely resident in the setup and calibration of PedBotHome in preparation to game-mediated exercising. A 13-year-old girl and her father recount their experience.

Dad: At first, I helped her, but when it’s working, it was fairly easy. Once it works, it’s easy to set up.

Daughter (age 13): After like 10 days, I started turning it on myself.

Dad: At times you forget to plug it in the phone [a cell phone was used as a component]

Daughter: I keep forgetting to charge the phone.

Dad: Yes, sometimes it’s the phone and you forget that you have to plug in the phone and charge it, but that’s a minor inconvenience. When you’re ready to do it, then you just plug it, so it’s somewhat charged. It’s fairly easy steps once it actually is working. It’s not complicated at all. You turn it on.

Complexity aligns with PYTHEIA item 5, ease of use (complexity, required effort).

##### Family Support or Disruption

Aligned. Family support took the form of parental structuring of time and the home environment. Interference with other activities was a source of frustration with PedBotHome.

Mom of 13-year-old girl: At first, it was a little difficult to figure out when we’re going to do it, how we’re going to do it [PedBotHome trial], how is the weekend going [to work out] because most of the time, I work on the weekends more than I do during the week. And so we had to figure that out. It took a little bit of working out because of work schedules and school and things like that.

Mom of 11-year-old girl. I think when they had an activity to get to, or they wanted to watch a show on TV, and she was expecting it [PedBotHome session] to be finished by, say, four o’clock and it would be finishing closer to 4:30, it started to get a little frustrating.

Family support aligns with both item 11, autonomy, and item 12, needing help from another person, on the PYTHEIA.

##### Fun Doing Exercise

Aligned. The waxing and then waning of the novelty of PedBotHome, and hence, how fun it was, was important to children’s appraisal.

Girl (age 11): I thought it was really cool for the first week. I thought it was the coolest thing ever. I would come home and I’d be like, “Oh, yes, I get to see PedBot”. Then towards the next couple of weeks, it got like, “Oh, I have to do PedBot today”. It was a really long thing. It made me not want to do it. I liked doing it once I got into it and once, I finished it and stuff, it was fun stuff. I wasn’t looking forward to it all day like I was in the beginning.

PYTHEIA Item 2, reflecting improvement to one’s everyday life, aligns with the concept of fun, particularly for a child.

##### Time Exercises Take to Complete

Aligned. Children were very conscious of the time spent completing PedBotHome exercises.

Boy (age 16). Every single time you fail, and then re-start, it takes a few minutes. So, it should be this 22-min [time] pressure this may be taking an hour, 40 min. I didn’t want to [invest that much time].

The PYTHEIA does not measure a time component and hence does not align with this factor.

##### Exercise Logbook

**Figure 4.**
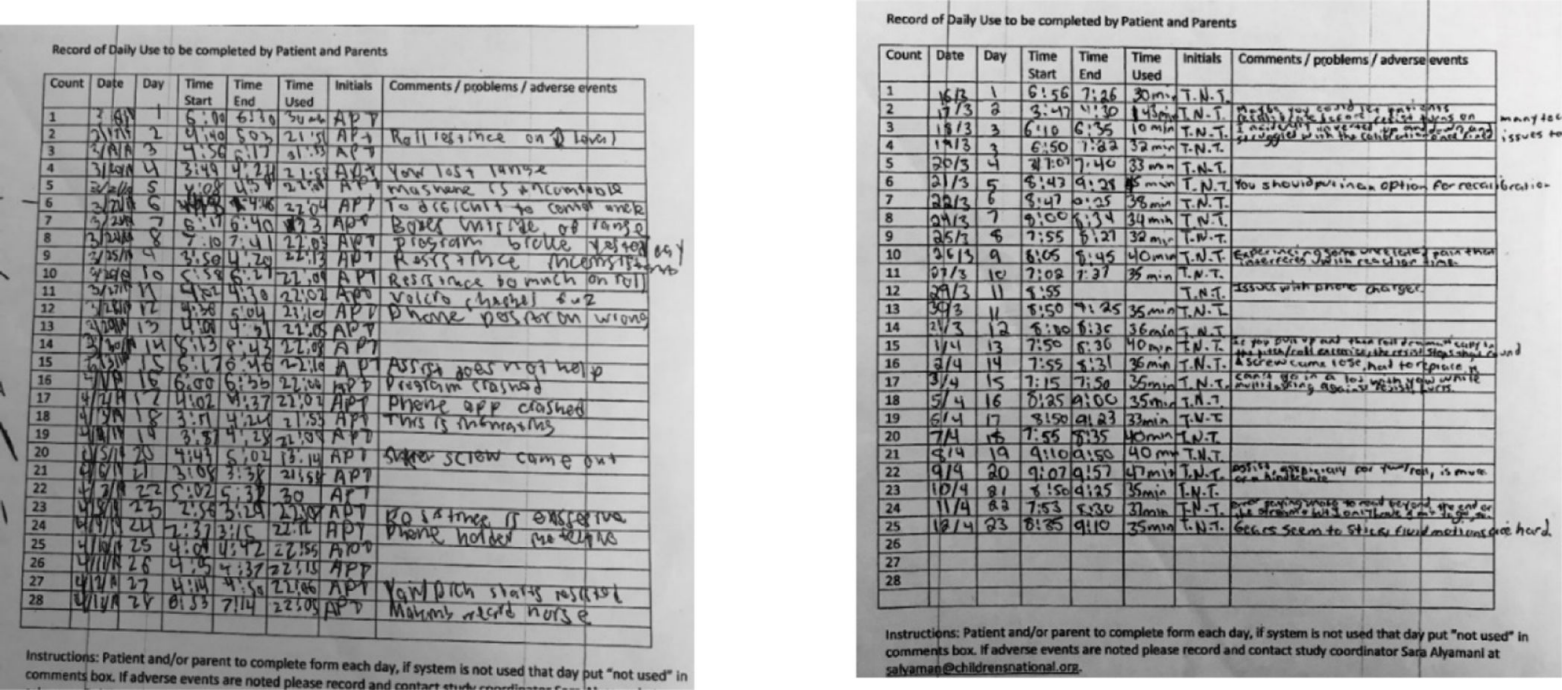
Detailed research logs kept by two children, age 16: boy, left; girl, right.

Ambiguous alignment. PedBotHome captures all data to the system so does not incorporate a logbook as is common in manual HEP. In the context of the research pilot, however, families kept a log of their use of theA/R gamebot and made notations about any problems they encountered. Some children found the logging satisfying. See Figure 4 for examples of detailed logbooks some children kept.

Girl (age 11): Actually, I really liked the logbook thing. I thought it was really convenient and cool. I thought that it really made sense to use it. I understood it really well, and I thought it was a good way to keep track of it, and that was the first thing that I did when I got into PedBot. I would write the date, and my initials and the time I started. I never missed something on the log. I thought the log was good.

A logbook function, manual or electronic, aligns with the PYTHEIA Individual Functionalities item set.

#### 3.1.2. Therapist Support

Not aligned (entire category). By design, it was exceptional for the study therapist to interact directly with families during the PedBotHome 28-day pilot. Consequently, none of the therapist support factors apply directly to the PedBotHome experience. The functions attributed to the therapist by Lillo-Navarro et al. [13], however, are essential functions and were carried out as a programmed function of the A/R gamebot technology or through support of family members and study software and hardware engineers.

##### Demonstrating Exercises

Training, including demonstrating the PedBotHome system, was conducted by the research technical team.

Girl (age 11): [First names of research hardware and software engineers] came while they were setting it up, and then I did my first round while they were there so if anything went wrong, then they would help me figure it out. They showed me how to get into the chair and how to strap my [foot in] and how to unstrap and how to turn on things. I didn’t really read the manual because I just learned from that one experience when they taught me how to do it.

Interviewer: Were you comfortable working with the engineers versus having a therapist there?

Girl: Yes. I thought it was totally fine. It was good. They were really helpful and stuff too, nice.

PYTHEIA item 12, needing help from another person, aligns and ease-of-learning items 3 and 4 may align with demonstrating exercises.

##### Coaching

Any need for troubleshooting was likewise addressed through voice or video calls from the study engineers.

Mom of 9-year-old girl: (considering) Problems where we had to call [first name of hardware engineer]

Daughter: Two, three.

Mom: Yes. Maybe three times. Something like that. It was usually because the Wi-Fi wasn’t connecting.

PYTHEIA item 12, needing help from another person aligns with coaching.

##### Perceived Regular Monitoring

Monitoring of data was performed regularly by the study therapist during the PedBotHome trial; however, families were neither aware nor concerned.

Girl (age 11). No. I didn’t know that they were watching. I didn’t know that it was a therapist because I thought it was just [software or hardware engineer] was watching.

Interviewer: Did you have any concerns that maybe the exercises weren’t right? You had the problem with the timing. Did you ever think that maybe the PedBotHome was stretching you too far, too long? Something that might have been allayed by therapists saying, “No. This is okay”.

Girl’s Mom: I’m fishing so hard. I think the answer’s no.

Girl: I don’t really know. I don’t think so.

No PYTHEIA item measures monitoring.

##### Giving Reminders

One family employed both direct and environmental reminders to their child with CP to interact with PedBotHome.

Interviewer: (to child) Did you need reminders to use PedBotHome at all?

Girl (age 11): Not really. I knew that my mom would be like, “Do PedBot now”, and I would be like, “Okay”. I always knew that I had to do it after school because there was a time built in for it. I never really needed someone to tell me to do it because it was right in the middle of where we do all of our stuff, so I would always see it and I would feel like, “I have to do it”.

Mom of Girl: Part of that was strategic. The hub of our household is our family room, kitchen, it’s one big space. So, I told them [the research team] definitely we want it right here because if it’s away from the action, [girl’s name] will have a hard time. She is not a kid that likes to be away from the middle of the action. She likes to be around everyone. If it were my older daughter who’s more introverted, I probably would’ve said, “Let’s put it off to the side somewhere because she likes that”, but that’s not [girl’s name]. It’s probably important for people to take into account the personality of the user a little bit. I think that helped for her to have it there, because then her little brothers would come around and be like, “Oh”. They’d be watching her do it. It was more interactive for her than being off somewhere by herself.

This family’s experience aligns with PYTHEIA item 1, adaptability, as well as items 11, autonomy, and 12, needing help from others.

##### Identifying Changes in Child’s Performance

Children thought of their performance as performance in the game. Progress in the game, a higher score, served as a proxy for therapeutic gains.

Interviewer: You’re nine years old, knew it was therapy. Were you thinking like, “I want to get a better score”. And then, “Oh. By the way, this is actually helping my ankle”. Or, “Heck with the score. I want to improve my ankle range of motion strength”?

Girl (age 9): I was thinking about the score.

Girl’s Mom: I think that’s true.

Item 2 of the PYTHEIA relative to improvement in one’s everything life aligns in both perspectives as short-term improvement, higher score in-game, and longer-term improvement, actual physiological improvement.

##### Providing Goal-Based Incentives

Again, the game score provided the child’s goal with in-game goals (scores) serving as a proxy for the underlying goal of physical improvement.

Interviewer: Did the game and your score, did that provide any incentive when you were interacting with the system?

Girl (age 9): A lot of the times I want to get higher scores.

Girl’s Mom: You would try harder.

Girl: Yes. Sometimes.

As above, item 2 of the PYTHEIA relative to improvement in one’s everything life aligns in both perspectives as short-term improvement, higher score in-game, and longer-term improvement, actual physiological improvement.

##### Providing Peace of Mind

Families were confident in the appropriate functioning of PedBotHome and did not need reassurance from the study clinician.

Mom of 9-year-old girl: Yes. I remember when we would do stretches and stuff and you just think, “I don’t know if I’m doing this right or enough”. Yes. It [PedBotHome] removed that completely. In some ways, you’re giving up control and so you’re just like saying, “Well, we’ll just see how this works”. Yes. It does.

There is no alignment between PYTHEIA measures and providing peace of mind.

Clinical, Regulatory, Engineering, and Commercial Product Domain Expert Advice on Role of Clinicians in Extending Therapy Provision Models

A business-to-business-to-consumer (B to B to C) model for engaging end users with novel rehabilitation technologies was advanced by the 24 experts we consulted. In this model, geared to the service delivery process in the U.S., therapists assume the role of early adopters of the technology, incorporating it into their practice and introducing their client/patient families to it. Families, subsequently, become secondary adopters.

#### 3.1.3. Personal Factors

All personal factors identified by HEP adherence theories aligned with the experience of adherence among PedBotHome families. Though the PYTHEIA measures the impact of technology on users’ autonomy and effort, it does not do the reverse, i.e., measure the impact of personal characteristics on technology acceptance. Since this is the case, the PYTHEIA does not align with the personal factors of HEP adherence theory listed below.

##### Autonomy

Aligned. Children demonstrated their autonomy using PedBotHome and parents endorsed and supported that characteristic.

Mom of 15-year-old Girl: Actually, almost every time I don’t have to be there at all. She does the whole thing by herself even [strapping her foot into the robot controller]. She can reach and do it”.

Mom of 11-year-old Girl: I think it’s easier for children to initiate it when they can be responsible for it when it doesn’t require a parent or some other caregiver being responsible to sit down with them. I think that one thing for busy families with other kids and lots of activities, it’s certainly nice when they can be responsible for it. I think she felt that way, too. She could take it over and didn’t have to wait for me or, “I have to run so and so here. I’ll be back”. Then, we’re delaying it. She could just come in from school and know her own schedule and do it.

##### Effort

Aligned. An 11-year-old girl describes the fluctuations in her effort showing the intertwined nature of effort, autonomy, and motivation.

Girl (age 11): I think that I put a lot of effort into it [piloting PedBotHome]. It depended, though. This is a little funny because sometimes, I was really into it and I really wanted to do really well on it. Then, other times, on the test, the 10-plane one, I would be like, “I really want to do good on this so that I can get a better score, and then they can all see how I’m improving”. It feels as something like every other one, I would have just been trying average or just okay. I wasn’t trying as hard as I did for the 10-plane. I think it really depended on which kind of run I was on.

##### Health

Aligned. Health issues limited some children’s adherence to the 28-day, PedBotHome regimen.

Mom of 13-year-old Girl: I know that we were supposed to do 21 [sessions] out of the month, [and] I really felt like we should be doing as much as we should do, but somedays, like when she was sick, I’m not going to force her to do whatever.

##### Motivation

Aligned. PedBotHome families described three different types of motivation to adhere to their exercise programs.

The perceived fun of the game framing PedBotHome exercise enhanced/impeded intrinsic motivation to engage in therapeutic exercise.

Interviewer: Any other thoughts about how the game could have been more motivating?

Girl (age 11): Maybe swapping out the games, making it more interesting. You could ask kids for opinions on games instead of just [deciding on your own].

The sense of purpose most families found in being a part of CP therapy research was another source of motivation.

Girl (age 15): Well, sometimes I get distracted. But I’ve tried to concentrate on the game.

Girl’s Mom: Like today for example she was looking into her phone trying to find when her French homework is due. She’s older she has more responsibilities and thinking about those things. Other than that, she thinks it’s really cool that she’s [involved]. She knows that not so many 15-year-olds get to have this opportunity to work in [technology research]. We’re very thankful.

The father of a 13-year-old girl reflected on the motivational trade-offs of having PedBotHome in the home versus using a similar system in the clinic.

Dad: When I drive her down there to [the clinic], she’s stuck. She has no other choice. She’s sitting in a chair, everybody’s around, you have no choice but to sit here till it’s done, and I’m sitting out waiting. When you’re at home, even though it’s convenient, it’s harder, in a sense, to use it. People think it’s easier in a sense, but it’s not always easy. You have to almost motivate yourself more to do.

##### Time Management

Aligned. Parents saw having PedBotHome in their homes as an efficient way to manage their own time better, avoiding time spent taking children to clinic appointments, while their children have more potential time in therapy given a home system.

Dad of 13-year-old Girl: It’s traffic going down there and coming back. That’s twice a week, so I had to readjust my own work schedule for that one, take some leave here and readjust work and all these other things. Having it here is a lot easier. Theoretically, [if] a person has it permanently in the house … let’s say a person uses it 15 min a day, four days a week, that’s 60 min. You can technically do more than any almost physical therapy because they have to schedule people to meet with somebody. That’s a whole other issue right there. When you’re at your home, you just jump on there for 15, 20 min a day, four or five days a week.

Children found the bug in PedBotHome where the system timer counted time in-game versus clock time particularly frustrating.

Girl (age 13): Sometimes it seems like when we put down the time and it’s 24 min, I feel like we’ve been in there for 35 or 40 min. So timewise, I think it would be better [to have the bug fixed], so we can gauge more. I think the right time would be a real great thing.

### 3.2. Alignment of PedBotHome Experience with Exergame Engagement Factors (RQ1b)

Five studies [35,36,37,38,39] reporting the exergame experience of children with CP together identified 17 engagement factors that divided across five categories: enjoyment overall, physical interface, game scenario and graphics, overall competence and control, and incentive from therapeutic awareness. PedBotHome experience aligned with 14 of the 17 engagement factors, did not align with two, and demonstrated ambiguous alignment with the remaining factor. See Table 5.

#### 3.2.1. Enjoyment Overall

##### Overall Degree of Game Enjoyment/Fun

Aligned. Novelty played a large role in children’s overall enjoyment of the PedBotHome exergame. An 11-year-old girl described the decaying of enjoyment over time.

Girl (age 11): I thought it was really cool for the first week. I thought it was the coolest thing ever. I would come home and I’d be like, “Oh, yes, I get to see PedBot”. Then towards the next couple of weeks, it got like, something that I came home and I was like, “Oh, I have to do PedBot today”. It was a really long thing. It made me not want to do it. I liked doing it once I got into it and once, I finished it and stuff, it was fun stuff. I wasn’t looking forward to it all day like I was in the beginning.

Item 2 of the PYTHEIA, improvement in everyday life, aligns with this factor.

##### Difficulty/Ease of Playing

Aligned. The difficulty and ease of play was principally associated with robotic footplate controller function but in-game design features played a role as well.

Interviewer: When resistance came on, how easy was it for you to do those exercises?

Girl (age 9): It mostly depends on the level of resistance and whether it’s that day, the resistance was working on that. Sometimes it did, but it didn’t always work.

Interviewer: Didn’t they used to have round hoops and they made them square?

Girl (age 13): I like the square hoops better. It’s easier to see how much you have to turn or lift your foot up or down.

Item 5 of the PYTHEIA, ease of use (complexity, effort) aligns with the factor globally as does item IF1, ease of use as applied to gameplay as an individual functionality of the overall system.

#### 3.2.2. Physical Interface

##### Range of Motion and Hold Time Diminish Fun

Range of motion and hold time corresponded to flexibility and strength-building respectively and were the focus of the therapeutic exercise designed into PedBotHome. The robot’s programming with respect to the current experience of the child in any given maneuver was developmental. A child explains how the lack of responsiveness of the robot detracted from her enjoyment of game activity.

Girl (age 11): Sometimes the assist and resist would make me uncomfortable sometimes because it would stay in one position when it would resist, and sometimes assist would feel like resist. It would resist I guess, and then it would stay in that spot. It got stuck, and then I couldn’t move it for one round and it would miss the playing for one round because I couldn’t use it, and then it would go back.

PYTHEIA individual functionalities items (IF1–5) align with controller experience.

##### Repetitions Do Not Diminish Fun

Ambiguous. Repetitive movements, such as repeated maneuvers to build up a score, were not remarked. The repetitiveness of the game itself detracted from the fun of the PedBotHome experience.

Girl (age 11): I got really annoyed with the airplane game because I thought it was just so simple and basic and easy and it was just—Not easy, but it was the same game and it didn’t make me want to do it every day because I was like, “I know what I’m going to do”.

Appraisal of repetitions, as a component of fun, aligns to PYTHEIA item 2, dealing with perceived improvements to life.

##### Game Controls Are Most Difficult to Get Positively Appraised

Aligned. A mother and daughter describe the difficulty with the PedBotHome control system.

Mom of 9-year-old-girl: Was it hard to do the exercises?

Daughter: Sometimes. It was frustrating. A lot of times, I get (gesturing up)… hard--

Mom: The plane would have to go--

Daughter: Go super high! and I can’t get [the controller to respond as fast as I needed it to]. It was frustrating.

Game control appraisal aligns with PYTHEIA individual functionalities items (IF1–5).

##### Being Comfortable While Playing

Aligned. PedBotHome was designed in consultation with clinicians to accommodate the various physical limitations typically accompanying CP. In addition to the discomfort experienced at the footplate controller, several children had difficulty positioning their upper bodies for optimal play.

Girl (age 16): It was really hard to get in and out of the chair, because there were like wires [all around] and [it was hard to] handle two things [getting positioned in the chair while avoiding the wires]. So, I found myself trying to get up [reposition continually]; [that put] a lot of stress on my left, until the time I really found myself [a new way] to stand up to get out of the chair.

Physical comfort factors do not directly align with PYTHEIA items though item 10, feeling secure, and item 2, improvement to everyday life, may pertain.

#### 3.2.3. Game Scenario and Graphics

##### Visual Esthetic

Aligned. Absence of pictorial variety was a uniform negative appraisal of the visual esthetic of the PedBotHome game.

Girl (age 13): It needs to be at least a couple of different pictures in there.

Her Dad: You can throw like maybe a space shuttle.

Girl: Or maybe like clouds in there. Seriously, [Name of software engineer]!

Dad: Different kinds of planes. These are programing things that they can look at.

Girl: Yes, more visual interest.

Visual esthetics may align as individual functionalities for evaluation in the PYTHEIA framework, as relates to everyday life experience improvement (IF-2).

##### Immersion in Game

Aligned. Immersion was important to children’s positive experience of PedBotHome. It was noted when immersion was broken, usually by an undesired response from the robot controller. A child describes how controller function distracted her from in-game higher-challenge flying tasks.

Girl (age 11): Most of the time when I went up and I went really far up, it would lock. My foot would lock up and then it would stay there, and then you had to do it a little, and then you just had to push apart to get it down because it would get stuck as you were going up.

Immersion does not directly align with any PYTHEIA evaluation factor, those it may indirectly relate to item 2 or item IF-2, if the game is perceived as an individual functionality, which measure experience of general life improvement.

##### Realism of Look and Feel of Game

Aligned. The graphics themselves met with general approval. Most children participating in the pilot of PedBotHome had prior experience of a more robust, clinic-based system, PedBotLab, which emerged frequently as a point of reference for the home-based A/R gamebot. However, the appeal of virtual reality (VR) with its greater native realism was noted by one child when prompted by the study therapist.

Girl (age 13): The graphics were fine.

Her Dad: The graphics didn’t seem to be different than the same game in [clinic location]. Playing the game looked the same, sounded the same. I didn’t see any difference.

Study Therapist: would you see a possible use for a VR system like this in the community health center? Because that’s increasing.

Girl: (Considering) I’d rather do VR? … I would rather do VR than this!

Interviewer: It’s always going to be your ankle. It would be VR, but you’d still be more immersed.

Dad: I’ve never done a VR thing. I’ve seen people do it, I’ve never done it.

Girl: It’s fun!

There is no alignment between realism as an evaluation factor and items on the PYTHEIA scale.

##### Enjoyment of Game Scenario

Aligned. Children’s prior experience with a more developed clinic-based system provided the concrete basis for wanting to interact with game scenarios they had enjoyed more while exercising. PedBotHome was designed in consultation with clinicians to accommodate the various physical limitations typically accompanying CP. In addition to the discomfort experienced at the footplate controller, several children had difficulty positioning their upper bodies for optimal play.

Girl (age 11): Yes. I did like the games that we did. I thought those were fun, but my favorite game was the horse game and that second wasn’t on PedBotHome.

Interviewer: The horse game. That’s on PedBotLab?

Girl: Yes. That was there, but it wasn’t at PedBotHome. I would need reminders to keep looking at the screen because, sometimes, I was just like, “Again, the airplane game? I really don’t want to do it”. I was just like, “They’re [her siblings] watching the TV”, and pretend I was doing it.

Enjoyment as an evaluation factor may align, with PYTHEIA items 2/IF-2, measuring life improvement.

#### 3.2.4. Overall Competence and Control

##### Sense of Competence Playing Game

Aligned. The importance of a sense of competence manifested in one younger child’s sense of satisfaction in beating the score of an older child on one of the bonus games packaged with PedBotHome.

Interviewer: So you did play the bonus game … was that good?

Girl (age 10): I would say yes. I like playing with the app.

Girl’s Mom: So the scores on the previous child, were on here. They were my … like, “That’s not supposed to be there!”

Interviewer: You beat [teenaged boy], you know how old [he] is?

Girl beams.

Competence does not align directly with PYTHEIA items. Partial alignment may be seen with item 11, autonomy and learnability items 3 and 4.

##### Sense of Control of Game

Aligned. Issues of control emerged from experience of the robotic footplate controller, as had been noted. PedBotHome suffered by comparison with the more developed clinical system. The assist function was designed to provide children a boost in performing more extreme maneuvers and its failure disappointed.

Girl (age 16). In the hospital the assist was great, but in this one the issue with the assist is, it is too slow.

Control as an explicit factor is not aligned with PYTHEIA items. Item 12, needing help from another person, is related to control.

##### Challenge of Game

Aligned. One child correctly identified the singular source of challenge, apart from therapeutically determined controller stiffness, as speed. She notes a need for other types of challenge to stay engaged.

Girl (age 15). The level of difficulty is like the same. Sometimes within a game it’ll get faster. So you have to put your foot in your right position faster as the level goes by. As the game goes on. How much you move your foot that part doesn’t really change. They could change it more often to get more playing.

No PYTHEIA item aligns with challenge.

#### 3.2.5. Incentive from Therapeutic Awareness

##### Perceived Therapeutic Function

Aligned. Parents and children both drew incentive to use PedBotHome from their perception that it was helping with functional performance.

Mom of 15-year-old Girl: I recall her climbing upstairs and it was way better than before. Her footsteps … even her personal trainer comment on how strong she got! I think [PedbotHome is] helping her with [sports], like [rock] wall climbing”.

Physical therapeutic effect does not clearly align with any PYTHEIA items.

Help Game Provides Correctly Doing Therapeutic Movements

Not aligned. Children perceived a greater therapeutic effect doing in-person, manual therapy.

Interviewer: Do you feel like the way the game was set up it actually helped you do your therapy correctly? Because you’ve done these rotations of your foot without the game, right?

Dad of 13-year-old Girl: Yes.

Interviewer: Did it feel like it was doing for you the same thing that [therapist’s name] was doing?

Girl: No, when I’m not in it, I can move my foot more around, and when I’m in there, it felt like my foot was in jail, the box around it. I think my foot was in a jail.

Dad: Jail foot.

No PYTHEIA item clearly aligns with help doing therapeutic movements.

##### Game Increased Motivation to Do Exercise

Aligned. The game made the prescribed exercises palatable.

Girl (age 10): It was fun. Because once you’re done with the whole entire thing you have games to play and even if you’re playing those games you’re still stretching your foot.

Interviewer: That’s a better way to discipline yourself to do that?

Girl: It’s like you want to [exercise without the game], but then you don’t. if it hadn’t been a game, I would have absolutely refused to do it.

No PYTHEIA items clearly align with motivation.

##### Game Spurred Child’s Initiative to Exercise

Not Aligned. This factor refers to exercise generally outside of the program incorporated in PedBotHome. No participating child currently had a prescribed HEP. All engaged in physical activities such as rock climbing, ballet, and yoga. There was no relation detected between A/R gamebot therapy and other physical activity. One mother describes her daughter’s situation.

Mom of 9-year-old Girl: We have been to [name of clinic] for physical therapy, but that was more than a year ago. It was more than a year ago, and maybe even two years ago. In between that and doing PedBot, we hadn’t done anything else. We’re trying to get [daughter’s name] to swimming classes and basketball after school, but other than that, no concerted effort.

Initiative and autonomy, PYTHEIA item 11, may be partially aligned.

### 3.3. Alignment of PedBotHome Experience with Acceptance Factors Measured by the PYTHEIA (RQ1c)

Of the 20 factors identified by the PYTHEIA for subjective evaluation of robotic technologies, 10 aligned with the experience of children and parents piloting PedBotHome, and two did not align. Two factors aligned, but with noted ambiguity, and further, two factors aligned but admitted at least a dual interpretation. The final four factors were of ambiguous relevance to the evaluation of an A/R gamebot technology as exemplified by PedBotHome. See Table 4 for a summary. Findings relative to alignment of PYTHEIA scale items with PedBotHome experience follow. (Note, main scale items are numbered 1–15; repeatable scale items, IF-1–5, are numbered 16–20).

#### 3.3.1. Adaptability

Aligned. PedBotHome placement within the family home involved deliberate choices on the part of parents and children. One mother strategically positioned the system at the center of family activities. See quotation, Section 3.1.2, 13 Giving Reminders. Another mother used the A/R gamebot to “seed” a therapy area for her 13-year-old daughter with CP.

Mom of 13-year-old Girl: I prefer to keep it out here rather than like on the living room or down the basement like I didn’t want to do that, I want her to be able to have a routine where that room could be where she does her therapy, she needs to stretch or anything like that with her little yoga mat or something.

Note that the scale item refers broadly to the “spaces where one spends one’s everyday life, home, work”. Our focus was only the home environment, given that home is central to the PedBotHome and the HEP it facilitates.

#### 3.3.2. Improvement to Everyday Life

Aligned, with a dual focus. “Improvement” mapped to improvements to the experience of doing exercise mediated by A/R gamebot technology. See comment associated with 16. Game Increased Motivation to do Exercise, Section 3.2.5 from the 10-year-old participant who stated that, had her stretching and strengthening exercises not been presented in a game framework, she would have refused to do them.

Improvement also mapped to the functional improvements that children experienced as a result of from doing therapy on PedBot Home. One child noted that she tripped less after spending time exercising using the system.

Girl (age 11): I used to be walking and then trip, and then walk and trip. Then since I’ve been doing it or when I was doing it, then I haven’t tripped and I’ve been walking more straight. I used to walk with my foot at a 45 degree/90-degree angle. Now I walk almost straight. It’s pretty straight. I think it [using PedBotHome] did make a difference.

#### 3.3.3. Ease of Learning All Individual Functions (Item 3)

Aligned, with ambiguity. Parents and children were at a loss to distinguish “all” individual from “basic” functions. Families focused on setup and run of PedBotHome as the most basic, essential functionality. The mother of a nine-year-old girl recounted an early experience demonstrating the importance of ease of learning the system.

Mom: It [PedBotHome] came. Then we had a day or two [using it]. Then I went away for a few days. Something had unplugged. My husband couldn’t figure out—or he didn’t try.

Daughter (age 9): (talking over) He couldn’t figure out.

Mom: how to put it back together. But then we finally figured it out when I got back. I thought it was pretty straightforward. It’s not complicating. That was easy.

#### 3.3.4. Ease of Learning All Basic Functions

See Section 3.3.3 above.

#### 3.3.5. Ease of Use (Complexity, Required Effort)

Aligned. See previous sections showing the importance of the complexity factor in both HEP, Section 3.1.1 item 5. Perceived Complexity of Doing Exercise, as well as exergaming Section 3.2.1 item 2, Difficulty/Ease of Play.

#### 3.3.6. Security

Ambiguous. Overlap with feeling protected, secure, confident (Section 3.3.10 below). The conversation with a nine-year-old girl and her mother pointed up the overlap among the PYTHEIA scale concepts: secure, protected, and confident.

Interviewer: (Talking about problems with the resist function of PedBotHome) Would you say that it interfered with your sense of security, but not necessarily your sense of safety? You didn’t feel unsafe, but you weren’t secure, you weren’t confident in the machine when it did that. Is that accurate?

Girl (age 9): Maybe. I never felt like anything was going to happen. I just felt, “It’s having a tantrum again”.

Mom: More frustration than any safety or insecurity?

Interviewer: You didn’t have confidence in it?

Girl: Yes.

Interviewer: Then actually the fourth question is about reliability. Is reliability [a] better [concept] than security?

Mom: Yes.

#### 3.3.7. Dimensions (Height, Width, Length)

Aligned. Families accepted the large footprint of the PedBotHome prototype in the context of research, but perceived it as in their way.

Mom of 9-year-old Girl: I wouldn’t say it was in the way, but it’s big.

Girl: It’s chunky.

Mom: Yes, it’s chunky, and the wires did get pulled out a few times. I’m not exactly sure how it all happened, but it was kind of right next to our computer and so, I think people would sit down with the computer and then inadvertently knock something over or whatever.

#### 3.3.8. Weight

Aligned. Families never moved the system but identified it as a concern in a non-research context.

Mom of 9-year-old Girl: We didn’t ever have to pick it up or move it. It didn’t really affect us. It’s heavy. If it were in our home [permanently], and we did have to move it …

Girl: It would be a two-man job!

#### 3.3.9. Sufficiency of Functionality

Aligned. The control interface and the game itself were the functionalities with which families were uniformly concerned. Refer to details in previous Sections: Section 3.2.2, Physical Interface; and Section 3.2.3, Game Scenario and Graphics.

#### 3.3.10. Feeling Protected, Secure, Confident

Ambiguous. See Section 3.3.6 above.

#### 3.3.11. Feeling More Autonomous

Aligned. Children demonstrated autonomy in the setup and use of PedBotHome (see Section 3.1.3, number 17, Autonomy) and this self-sufficiency aided their completion of sessions on the platform. Parents valued their children’s autonomy as an aid to managing their own adult schedules. This autonomy further promised a decoupling from the burden of coordination of and transportation to therapy appointments. The father of a PedBotHome family living in the far suburbs described.

Dad of 13-year-old Girl: There’s huge market as far as rehabilitation I think, especially in the outlying areas of [name of state], and once you get outside of [near suburbs] and closer to [the center city], but once you start going up to the [far suburbs], you don’t want to drive there [back into the city for where the clinic is]. I think a lot of people probably won’t even drive down to [the center city] just because you can’t spend three hours coming back. It’s brutal, it really is. We actually did the [commuter train] one day, it’s just as bad. It was just bad, even though you’re not driving. … [So] yes, as far as [doing therapy] remote-wise, absolutely. In the future, I think if the system [PedBotHome prototype] was tight, yes, you’d save having to go down there. You see a lot of people going down there, hours are spent driving [that could be used for something else].

#### 3.3.12. Needing Help from Another Person to Use

Aligned. This item is related to Section 3.3.11, feeling more autonomous. Children did not typically need help from another person, after initial setup, to use PedBotHome. However, several did not endorse the value of disconnecting from another person for the sake of not requiring help. (See Section 3.2.5, number 15, Help Game Provides Correctly Doing Therapeutic Movements.) An 11-year-old girl recollects her experience, from several years earlier, with a physical therapist and her mother supporting her in practicing exercises at home.

Girl (age 11): Yes. I think that it was a lot easier to do regular exercises. I don’t really remember as much, but I feel like it’s a lot maybe more effective. When we went to view my rechecking not all of my things improved, but I feel like if I did something like regular exercise every day, then maybe it would improve better because it would be—not just in one spot. I’d be able to move around enough. I think if I use regular exercises, it was more free, I guess because I had the freedom to walk around I guess. Since I’ve been doing PedBot, and PedBot you’re pretty much just sitting down the whole time. I feel like PedBotHome was easier, or it was harder to do that because it didn’t give me all the strength in all of my muscles. It was just a little bit my foot muscles pretty much, and I feel like if I did exercising it would be stretching out all of my muscles.

#### 3.3.13. Comfort Using in the Community

Not aligned. The device is designed exclusively for home use.

#### 3.3.14. Comfort Using around Colleagues (Working Environment)

Not aligned. The device is designed exclusively for home use.

#### 3.3.15. Comfort Using around Friends and Family

Aligned. Using PedBotHome conferred a “celebrity” status on children in the pilot. Two girls recounted how their family and friends positively affirmed them when they demonstrated exercising on the system.

Girl (age 9): There was one friend who loved it [PedBotHome]. She would literally get a snack from the pantry and just watch me do it. She said it was like she was eating popcorn while watching a movie”.

Mom of 11-year-old Girl: Anybody who came over would ask about it, and we were excited to share about it. The other kids, we have four kids, and so all of them were fascinated by it. It was fun. It was something that I think was a really good experience overall.

Daughter (age 11): Yes, I think I agree with you, mom. I think it was like when people came over, they were interested in it and they would watch me do it and be like, “That’s really cool”.

#### 3.3.16. Individual Functionalities, Item IF1, Ease of Use

See analogous item Section 3.3.5 above.

#### 3.3.17. Individual Functionalities, Item IF2, Help Provided in Everyday Life

See analogous item Section 3.3.2 above.

#### 3.3.18. Individual Functionalities, Item IF3, Safety, Security

See analogous item Section 3.3.6 above.

#### 3.3.19. Item IF4, Reliability

Aligned. The expected breakdowns that occurred in testing the PedBotHome prototype in children’s homes pointed up the important of reliability as a use factor. The father in one family that experienced difficult-to-resolve system failures during the trial described the interaction of reliability and family routine.

Daughter (age 13): If it stops working, you don’t use it.

Dad: It’s like a game, if you don’t use it, it’s going to be, “No, I’ll try again tomorrow”. You turn it on and see if it works. I think that’s important because if it’s at home, you schedule. You create a little time to do it, but in the scope of things in a day, you have other things to do.

Interviewer: So it needs to be on when you’re ready.

Dad: Yes, that’s a big thing.

#### 3.3.20. Item IF5, Feeling of Safety

See analogous item Section 3.3.10 above.

## 4. Discussion

### 4.1. HEP Adherence Theory, the Experience of PedBotHome, and PYTHEIA Scale Alignment

#### 4.1.1. Domain: Fit of Exercise in the Home Environment

In the domain of fit of the HEP program into the home environment, PedBotHome families experience was very well-aligned with HEP adherence theory developed in the pre-robotic context. PedBotHome’s internal, electronic exercise log, analogous to a pre-robotic handwritten log that families would use for tracking, did not enter into the experience of our families. Rather, we asked participants in the PedBotHome pilot to keep a research log to help identify bugs and other issues. Two children were scrupulous in keeping this log (Figure 2) and presented it to the research team with pride. Another child told us she really enjoyed keeping the handwritten log. Though handwritten recordkeeping in conjunction to use of a digital system is counter-intuitive, the idea warrants further investigation. Handwriting has been shown to trigger neural pathways for learning in ways that that keyboarding does not [41] and digital ink may provide a similar benefit without recourse to traditional pen and paper. Evaluation of a potential child log functionality aligns with the PYTHEIA IF subscale.

Several areas of variance of PedBotHome experience with the PYTHEIA scale came into focus based on exploration of the Fit of HEP in the Home Environment domain. The factor “Perceived Effectiveness of Exercise” aligns well with what would be a *prima facie* understanding of PYTHEIA scale item 2 dealing with the improvement experiences in one’s everyday life as a result of using the target technology. The adherence factor, “Fun Doing Exercise”, however, suggests that PYTHEIA scale item 2 is open to multiple layers of interpretation that should be clarified. An exercise can be fun but not effective. The distinct is temporal: short-term versus longer-term improvement in one’s life. This is a distinction that is relevant to rehabilitative technologies, RT, that is not relevant to assistive technologies, AT. Both technologies are instrumental, used to achieve a goal beyond use of the technology. In the case of RT, the use may or may not be enjoyable in the short-term, but the user may persist because of the improvement to life anticipated in the long-term. One child in the PedBotHome pilot remarked that she believed that traditional exercise was better for her, this apart from whatever fun an A/R gamebot such as PedBotHome might provide (See Section 3.3.12).

The HEP adherence factor, “Comfort during Exercise”, is not clearly aligned with the PYTHEIA scale. Comfort impacts quality of life, suggesting some coverage by scale item 2, life improvement. The protected concept embedded in scale item 10 also pertains to comfort. Notably, the PYTHEIA scale incorporates three items, 13–15, focused on social comfort but neglects physical comfort, which would seem to be the principle thrust of the HEP adherence factor, given that the context for all HEP theory is strictly the home, with the intrinsic privacy it provides and protection from social exposure.

The possible linkage between the adherence factor, “Family Support or Disruption”, and PYTHEIA scale items is complicated by the fact that any evaluation of a system aimed towards children necessarily has to take the perspectives and priorities of decision makers, i.e., parents, into account as well. Parental support and tolerance for disruption was strongly tied to the concepts of autonomy and needing help from another person, PYTHEIA scale items 11 and 12. Though parents apparently valued their children’s autonomy as an intrinsic good, they also valued it as an aid to their own coping and life organization (See Section 3.1.3). The most universal positive parents cited relative to PedBotHome was the degree to which it would free them from the inefficiency of taking their children to therapy appointments, which in the PedBotHome catchment area almost always entailed lengthy commutes (See Section 3.3.11). Children’s investment in autonomy and not needing help from another person to carry out HEP was of a different quality. Two children in particular were tentative about losing the interaction with their therapist that the successful integration of PedBotHome implied (See Section 3.2.5 and Section 3.3.12).

“Time Exercise Takes to Complete” was an important adherence factor not covered by PYTHEIA scale items. The lack of a specific time-based component of evaluation in the PYTHEIA may, again, reflect a difference in expectation of RT versus AT. One expects to stop activity, use RT to accomplish a therapy goal, and then resume activity without the RT. On the contrary, one uses AT to facilitate activity. It is incorporated into the activity. When AT starts, the desired activity starts. When AT is put aside, the desired activity is likewise put aside.

#### 4.1.2. Domain: Therapist Support

Since one of the goals of A/R gamebot technology is to enhance clinician efficiency and effectiveness by automating some of the more routine and repetitive tasks of therapy, none of the interactions with (specifically) the child’s therapist found to be important to HEP adherence in a pre-robotic era program aligned with the experience of PedBotHome. This is not to say that the supportive activities that therapists have typically carried out in conventional, pre-robotic HEP did not align. In the case of PedBotHome, these activities were important but carried out by research technical staff or this child’s parents. The therapist monitored the child’s progress behind the scenes and conducted physical assessments pre- and post-PedBotHome intervention.

There was alignment between the supportive factors per se, identified with the therapist in HEP adherence theory though carried out by others in the PedBotHome pilot, and some PYTHEIA scale items. There was no alignment between the supporting factors “Perceived Regular Monitoring” and “Providing Peace of Mind”. As noted with respect to the logbook in the previous section (Section 4.1.1), regular monitoring might be accomplished via a logbook function and measured using the PYTHEIA IF subscale.

Important supportive functions such as “Demonstrating Exercises” and “Coaching” align with PYTHEIA scale item 12, needing help from another person, and items 3 and 4 that deal with technology learnability”. Giving Reminders”, as experienced by parents and children piloting PedBotHome, aligned, perhaps unexpectedly, with PYTHEIA scale item 1, measuring the adaptability of the technology to the target environment, as well as with items 11 and 12, whose focus is autonomy and needing help from others.

The supporting factor, “Identifying Changes in Child’s Exercise Performance”, aligns with PYTHEIA scale item 2, focused on life improvement. Interestingly, this change was largely appreciated through the proxy of change in the child’s game score, if not through self-perceived improvement in gait and other physical activities.

#### 4.1.3. Domain: Personal Factors

All personal factors identified by HEP adherence theory align with the experience of PedBotHome families. None, however, align with PYTHEIA scale items since the directionality of each is reversed. For example, according to the HEP adherence theory, the personal characteristic of autonomy predisposes a child to adhere to his/her HEP. In the PYTHEIA framework, conversely, autonomy is measured as an experience conferred through use of the target technology. Experience of the pilot demonstrated that more autonomous children had better adherence to the testing protocol. Doing PedBotHome did not confer any perceptible autonomy on participating children over the 28-week period of their engagement with the A/R gamebot.

Given that theory identifies five personal factors, Autonomy, Effort, Health, Motivation, and Time Management (skills), as important to HEP adherence and that our small sample, likewise, associated these characteristics with adherence to the pilot protocol, further thought should be given as to how they might be incorporated into interpretation of the experience of A/R gamebots for both design iteration and clinical implementation.

### 4.2. Exergame Engagement Theory, the Experience of PedBotHome, and PYTHEIA Scale Alignment

As was the case for HEP adherence theory, the experience of PedBotHome aligned well with engagement factors previously identified in the literature with a focus on children with CP. For children, game objectives occupied the evaluative foreground and sometimes obscured the background, therapeutic objectives for them. This situation was most pronounced when the goals of therapy (i.e., increasing strength and range of motion through increased controller resistance) impeded the goals of the game (i.e., increased resistance made the child miss a target) (See Section 3.2.2). The games used within an A/R gamebot and the system’s controller, hardware, and software components respectively, appear amenable to evaluation as individual functionalities on the PYTHEIA IF subscale.

#### 4.2.1. Domain: Overall Enjoyment

Both factors categorized as pertaining to overall enjoyment, “Overall degree of game enjoyment/fun” and “Difficulty/Ease of Playing”, aligned with PedBotHome experience as well as with the PYTHEIA scale. Item 2, contribution to improvement in everyday life, pertains. The qualification described in the previous section applies to the game as well as the exercise. It is the immediate in-game experience that pertains, not the derivative functional improvement anticipated by a game-used-seriously. As also previously noted, since it is possible for the perceptions of immediate improvement resulting from game enjoyment and the longer-term improvement in function to diverge, the context of improvement needs to be clearly specified for the user rating experience.

#### 4.2.2. Domain: Physical Interface

All physical interface factors aligned with PedBotHome experience except for the contention that repetitions did not diminish fun. Clarification is needed. The repetitive ankle motions required to maneuver the plane in PedBotHome’s principal flying game may have been the subject of complaint because of perceived imprecision or malfunction versus having to do them (with a bit of discomfort from added therapeutic resistance) as repetitive actions. Repetition in terms of the graphical interface was clearly disliked, however (See Section 3.3.2, number 4). Our experience in PedBotHome strongly affirms Whittinghill and Brown’s contention that game controls are most difficult to get positively appraised [36].

Evaluation of the game controller as an individual functionality maps clearly to the PYTHIA IF subscale. The factor of comfort arises in the exergame engagement paradigm as it did in the theory guiding HEP adherence. We observe again that physical comfort is not clearly measured in the PYTHEIA scale, though partial alignment with items 2 (improvement to life) and 10 (feeling protected) can be made.

#### 4.2.3. Game and Scenario Graphics

All identified game and scenario graphics align with the PedBotHome experience. The most common problem related to “Visual Aesthetic” was lack of variety and visual interest. “Immersion in the game” was impeded by control issues as well as by distraction. “Realism of look and feel of game” was not an immediate issue though one child negatively compared to game to VR (See Section 3.2.3, number 9). The concept of realism did not seem well aligned with any measure in the PYTHEIA scale. The other game and scenario graphics factors, with proper framing, could potentially be measured by the improvement to life PYTHEIA scale items, particularly if framed as IF subscale functionality. When we are evaluating visual components of an immersive technology, we are very far from robotics and rehabilitation, even though those components are present in a robotic rehabilitation system like an A/R gamebot. Future work may want to investigate integration of items measuring the flow state [42] adapted to exergaming and A/R gamebot technology.

#### 4.2.4. Overall Competence and Control

PedBotHome experience aligned with all three identified competence and control factors. Mirroring of these factors in the PYTHEIA was ambiguous or seemingly absent. Challenge, in particular, is absent from the PYTHEIA scale. This absence again points up the difference between AT and RT. “Challenge” in AT makes no sense. The purpose of AT is to compensate and reduce challenge. Challenge is part of therapy, however, both in the physical and mental sense. In an A/R gamebot, the challenge of the game mediates the underlying physical challenge to exercise and push boundaries. A sense of competence is implied in PYTHEIA items 3 and 4, dealing with learnability (mastery) and item 11, autonomy. An echo of “control” is partially heard in scale item 12, needing help from another person.

#### 4.2.5. Incentive from Therapeutic Awareness

Children, the youngest of whom was 9 years old, were aware of the underlying therapeutic purpose of PedBotHome, and it contributed to their motivation to exercise. Because of many issues experienced with the footplate controller, children were not convinced that the A/R gamebot provided them better therapy than they could do freeform. There was no indication that using PedBotHome spurred children to take on further exercise programs outside of the pilot. Many were engaged in other activities, but the connection between those activities and PedBotHome did not emerge.

From an AT perspective, therapy is not generally germane and we do not see an appreciation of the strictly therapeutic in the PYTHEIA. None of the first three factors in the Therapeutic Awareness domain—“Perceived therapeutic function”, “Help game provided correctly doing therapeutic movements”, and “Game increased motivation to perform exercises”—align. That said, the “initiative” aspect of the fourth factor, “Spurred child’s initiative to exercise”, aligns well with PYTHEIA item 11 measuring autonomy, with the appropriate directionality (i.e., using the technology increases initiative/autonomy). In this case, the therapeutic motivation is irrelevant.

### 4.3. The PYTHEIA Scale: What Works for A/R Gamebot Assessment and What Might Be Improved

#### 4.3.1. The Individual Functionalities (IF) Subscale and Evaluation of Functionality in General

The PYTHEIA IF subscale holds a lot of promise for conducting increasingly granular inquiries into user experience at the system component level. Table 6 lays out an exercise to think about, first, evaluating a child’s experience of the PedBotHome robotic footplate controller and, second, the Airplane Game that currently provides the backbone framework for delivering the child an appropriately therapeutic dosage of ankle stretching and strengthening exercises. We step through the evaluation simulation below.

- Item IF1 queries ease of use. This item is clear, whether the target functionality involves hardware or software.
- IF2 queries the help the specified functionality provides “in your everyday life”. Here an interpretation is needed. Two possible interpretations relative to the footplate are (1) how well is supports functional improvement by delivering appropriate exercise or (2) how well it works in controlling the game. These two “helps” may or may not align. Similarly, with respect to the airplane game, two possible ways it could be helpful in everyday life are (1) how generally entertaining the game is or (2) how much it helps engage the child in his/her HEP. These two options likely converge, but not necessarily. The question “Help what?” needs to be explicit to be sure all users are evaluating the same phenomenon.
- IF3 deals with safety and security. As noted, these factors are ambiguous in the context of a device such as an A/R gamebot. We know the hardware caused discomfort for some children in the PedBotHome pilot and this problem may map to IF5, the feeling of safety. It may also map to IF4, reliability: insecurity secondary to unreliable functioning. The item does not resonate when applied in the realm of software, i.e., The Airplane Game. The response in this latter case would be N/A.
- IF4, reliability, applies equally well to our selected hardware and software functionalities.
- IF5, finally, may make sense in the case of hardware strapped onto a child’s ankle but it does not in the case of software.

What is concerning in this mockup is that the software component would end up with two out of three components missing, suggesting a threat to the validity of the final evaluation. Defining different evaluation items for hardware and software functionalities, effectively different subscales, may be a useful approach. Notably, the main scale of the PYTHEIA also has items (3) dealing with functionality. As noted in Section 3.3.3, families were at a loss to enumerate the total functionality (item 3) of PedBotHome, and then decide which of those functions were basic, concerned them more (item 4). It was much easier to identify what seemed missing, functionality they would have liked but did not have (Item 9). It might be helpful to administer the IF scale(s) first and the main scale subsequently. Proceeding in that order would allow users to think about each functionality as they evaluated them and have that recent evaluation in mind when they subsequently encountered the three functionality questions in the main scale. Otherwise, there is a risk that different individuals will be thinking of different functionalities as they complete the main scale items.

#### 4.3.2. Personal Factors

Taylor et al. [13] identified personal factors as one of the two pillars of HEP adherence in CP. (See Figure 3) We noted these same factors in our extremely adherent (to HEP in the research context) participant families. These factors are highly likely to influence subjective appraisal of A/R gamebot technology. The PYTHEIA does not incorporate personal factors, though some in the AT tradition on which it builds (such as Scherer et al., in their Matching Person and Technology framework [29]) do. It may be helpful to consider personal factors as we move forward in instrument design and testing.

A question that flows from thinking about personal factors is that of identifying the class or classes of persons to whom we will target an A/R gamebot assessment instrument. Parents and children with CP are both stakeholders in system use; however, their interests diverge. As we noted especially in the discussion surrounding autonomy, the concept means different things to parents and children. Parents consider how a system like PedBotHome impacts their own autonomy as well as that of their children. Children only see their own interest in this particular case. One of the decision we will make in the next phase (design) of instrument development is how we will align scale items with parent versus child users. For example, we may score items differently for parents and children, or we may construct separate parent and child subscales. It seems clear from this study that assessment of one without the other (i.e., parents or children alone) will provide a very incomplete picture of how a system is accepted in the home.

#### 4.3.3. Short-Term versus Longer-Term “Life Improvement”

PYTHEIA main scale item 2, which is essentially the same as item IF2 in the IF subscale, similarly requires clarification of “Improve what?” in everyday life. The question asked with respect to the system as a whole admits the same ambiguity as it does when asked with respect to subfunctionalities. It is especially important to clarify what level of outcome the item targets: whether the user is being asked how much the A/R gamebot improves his or her ability to adhere to therapy or whether the A/R gamebot results in perceived functional improvement, the ultimate goal of engaging in therapy. There is an added level of instrumentality. Increased adherence, in theory, leads to increased functional improvement. This issue of an added layer of instrumentality emerges in technologies that are rehabilitative (RT) but under the control of the user/patient. Technology that goes home with the patient has typically been assistive technology, which has a different use case from RT. Though there is certainly an esthetic component to AT acceptance as well, it is hard to imagine a person rating a wheelchair with no wheels (i.e., dysfunctional) highly, no matter how shiny. It is not at all hard to imagine a child enjoying an A/R gamebot with an engaging scenario where s/he racks up thousands of points and develops a sense of mastery but sees little to no functional improvement in the exercising limb.

#### 4.3.4. Assistive Technology (AT) Operating at Two Conceptual Levels

It seems we must either give up the notion that there is such a class of things as A/RT (assistive and rehabilitative technology), or we must differentiate AT in the context of life (the traditional perspective, in the service of ADL—activities of daily living) from AT to achieve a rehabilitative purpose, such a carrying out therapy at home. If we adopt the latter approach, A/R gamebots remain legitimately assistive and the “A/” part of the name can stay. RT has been framed, in the context of the International Classification of Function [43] as an “indirect” AT [44]. Rather than directly mediating augmentation of function through compensation (as is the strategy, for example, of screen readers relative to vision and wheelchairs relative to mobility) RT indirectly supports the individual’s function by assisting in restoring the target functional deficit. When the client possesses and controls the RT (such as PedBotHome), some evaluation criteria applicable to traditional AT systems apply, and others do not. As also seen in authors’ parallel efforts focused on robotic systems mediating user-controlled hand rehabilitation after stroke [32], families’ experience with PedBotHome pointed up key differences between rehabilitative technologies used to assist patients in taking a more active role in their own therapeutic exercise to intensify impact and technologies whose primary function is to assist in activities of daily living. That user-controlled RT and AT as typically conceived are not identical paradigms will be an important point in developing an evaluation schema for A/R gamebots, which may incorporate features of both the RT and AT paradigms. It fact, most of the items in the PYTHEIA that did not align with the experience of PedBotHome did not do so because of the underlying assumption that the technology under evaluation, albeit robotic, was framed by a predominantly AT use case.

#### 4.3.5. Referents in the PYTHEIA That Do Not Align with Assistive Technology for Rehabilitation Conceptually

The three principal contexts carried by PYTHEIA scale items that malaligned with evaluating the PedBotHome experience were (1) the focus on safety and security (items 6, 10, IF3, an IF5), (2) the projection of technology use beyond the home (items 1, 13, and 14), (3) adult versus pediatric technology use (items 1 and 14). This might be remedied by allowing the user to pick a context of use. There is also an underlying presumption that the technology will “travel” with the individual, as might be expected in the traditional AT context. Picking the environment would fix that ambiguity.

#### 4.3.6. PYTHEIA Items to Carry Forward

Scale items focused on evaluation of static physical (i.e., dimensions, weight) and dynamic (i.e., reliability, ease of use, learnability, sufficiency of functionality) will be carried forward from the existing validated PYTHEIA scale to the emerging A/R gamebot instrument. Items focused on the home (i.e., adaptability, comfort using around family and friends) will also be carried forward.

#### 4.3.7. Potentially Important Use Factors the PYTHEIA Does Not Measure

Factors the PYTHEIA does not measure that emerged from plotting the PedBotHome experience against the framework of CP HEP adherence theory and exergame engagement were fun/enjoyment, comfort, time commitment, clinical monitoring (perhaps aligned with the PYTHEIA scale security items), and challenge. We will consider these factors in the next phase of instrument development where we will generate a candidate item set for cognitive and, subsequently, pilot testing.

Implicit in CP HEP adherence theory, though not in theories of exergame engagement, is the understanding that perceptions formed around therapeutic activities composite those of multiple actors, at a minimum, those of the child with CP and his/her parent. Beyond the known parameters of the robot-controlled computer game that mediates a child’s therapy, there is a quasi-*game theoretic* scenario playing out between parent and child. Game theory deals with situations characterized by both cooperation and conflict and is beginning to be used to model interactions in health care [45]. PedBotHome parents and children demonstrated overlapping interests in the technology, but weighted those interests differently. Children focused on play and therapeutic value; parents focused on therapeutic value and convenience. Each actor may be expected to form his or her utility function, the decision to adopt or reject an A/R gamebot system, accordingly. This observation suggests a need to develop items that target parents and children separately and evaluate how each contributes to a go/no-go assessment of A/R gamebot technology.

## 5. Limitations

This study has several limitations. As is typical of other work in technology development focused on the needs of children with CP, our sample size was small. (See for example, the 16 studies retrieved from the systematic review of games used seriously in CP conducted by Lopes and colleagues [21]. Six of these efforts reported sample sizes in the single digits, and only one reported on a cohort of more than 20 children.) There was also a disproportionate number of girls versus boys (7:1) who volunteered to participate in the pilot of PedBotHome. Gender is likely to have a skewing effect on acceptance of the system. The protocol asked a high commitment of time from our families, and individuals with unusually strong motivation are likely to have self-selected, creating another opportunity for introduction of bias. Finally, study engineers (the researchers directly involved in system design and implementation and in most frequent communication with participants) were within earshot during several of the interviews. Children’s and parents’ concern for the feelings of the people most intimately involved in the workings of PedBotHome may have impeded their candor.

## 6. Conclusions

As we move forward with the development of items for subject assessment of A/R gamebots, we will be guided by several key principles that have been suggested by this conceptualization study. Most fundamentally, we will proceed mindful of the need to clearly differentiate the assistive and rehabilitative use cases and how each applies in the case of guiding users to consider their A/R gamebot experience. Also essential to item crafting is the understanding that successful gameplay, for the child, largely mediates perceive success of the therapeutic process. Enjoyment of interacting with the system, however, is distinct from, and in tension with, perceived effectiveness relative to clinical outcomes. Finally, the user evaluation that is meaningful is one that integrates the perceptions of both the child with CP who actually interacts with the system and those of his/her parents (or guardians), who essential players in decision making around A/R gamebot adoption and use.

## 7. Patents

US20200038703A1: Robotically assisted ankle rehabilitation systems, apparatuses, and methods thereof. https://patents.google.com/patent/US20200038703A1/en?oq=US20200038703A1.

## Supplementary Material

Codebook - Adherence Theory - 8-31-2020

PYTHEIA Items and Scale Formats

## Figures and Tables

**Table 1.** Conceptualization of an instrument for the subjective evaluation of A/R gamebot technology.

Logic Model: A/R Gamebot Subjective Evaluation Instrument Conceptualization		
Activity	Inputs to Instrument Conceptualization	Outputs Identified for Initial Item Generation
Literature Review	Horne Exercise Program Adherence Theory	
	Games Used Seriously—Theories of Use and Effect	•Target Variables (Items): What factors are essential to the measurement A/R gamebot acceptance?
A/RT Instrument Review	PYTHEIA—Validated Scale for Measuring Explicitly Robotic Assistive and Rehabilitative Technologies	• Inclusion/Exclusion Criteria: Whose acceptance pertains (what population(s) should the instrument target?)
Family User Interviews	Eight families (9 children, eight parents) participating in 28-day home pilot of PedBotHome A/R Gamebot prototype	•Technology Use Cases: To which use cases will the candidate items apply?
Clinical, Engineering, Regulatory Expert Consultations	In conjunction with grantor-sponsored program to explore mechanisms of technology transfer	

**Table 2.** PedBotHome participant demographics.

Participant	GMFCS Level	Age	Sex	Facilitating Parent	Number of Days Played (Out of 28)
1	2	15	female	mother	27
2	1	13	female	mother	19
3	2	16	male	father	28
4	2	16	female	father	24
5	2	10	female	mother	5*
6	1	9	female	mother	21
7	1	11	female	mother	17
8	2	13	female	father	7 +

**Table 3.** Home exercise programs (HEP) adherence theory alignment with PedBotHome experience and PYTHEIA [31] scale items.

THEORY 1 [12]	THEORY 2 [13]	Adherence Factors (Synthesis)	Alignment with PedBotHome Experience	Alignment with PYTHEIA Scale Items
	**Fit of Exercise Program in the Home Environment**			
x		Exercise Equipment	Aligned	Aligned. Similarly, the technology itself is focal.
x		What the Exercise Is	Aligned	Alegned. Responsive to Individual Functionalities flexibility.
	x	Perceived Effectiveness oe Exercise	Aligned	Aligned. Contribution to improvement to one’s everyday life (item 2).
	x	Comfort During Exercise	Aligned	Not clearly aligned. Potentially implicit in items 2 and 10. Items 12–15 deal with specifically social comfort.
	x	Perceived Complexity of Doing Exercise	Aligned	Aligned. Item 5, ease of use (complexity, required effort)
x	x	Family Support or Disruption	Aligned	Aligned. Item 11, autonomy and I tem 12, needing help from another.
	x	FunDoing Exercise	Aligned	Not clearly aligned. Results from ambiguity of interpretation of item 2, improviment to everyday life. Need to distinguish short-term improvement (inâme fun) from longer-term, therapeutic outcome improvement.
x	x	Time Exercises Take to Complete	Aligned	Not aligned. No explicit time component in the PYTHEIA.
x		Exercise Logbook	Ambiguous	Further exploration needed. PedBotHome system log not presently accessible to the user. Conceivably, a logbook function could be an Individual Functionality.
		**Therapist Support**		
x	x	Demonstrating Exercises	Not aligned	Aligned. Item 12, needing help from another; implicitly, the ease of learning items, 3 and 4.
x	x	Coaching	Not aligned	Aligned. Item 12, needing help from another; implicitly, the ease of learning items, 3 and 4.
	x	Perceived Regular Monitoring	Not aligned	Not aligned. No items measure monitoring.
	x	Giving Reminders	Not aligned	Aligned. Item 1, adaptability. Items 11 and 12, autonomy and needing help respectively.
	x	Identifying Changes in Child’s Exercise Performance	Not aligned	Ambiguous. Item 2 pertains but improvements identified NOT through therapist but through change in game performance (score) and/or subjective experience in physical activity.
	x	Providing Goal-based incentives	Not aligned	Ambiguous. As immediately above.
	x	Providing Peace of mind	Not aligned	Not aligned
		**Personal Factors**		
x		Autonomy	Aligned	Not Aligned. Item 11, autonomy, has opposite directionality.
x		Effort	Aligned	Not aligned. Item 5, ease of use (complexity, required effort) pertains to interaction with the device, not to personal satisfaction in putting forth effort.
x		Health	Aligned	Not aligned. Health factors not addressed by PYTHEIA scale.
x		Motivation	Aligned	Not aligned. Personal factors not addressed by PYTHEIA scale.
x		Time Management	Aligned	Not aligned. No explicit time component in the PYTHEIA.

**Table 4.** PYTHEIA [31] scale items alignment with PedBotHome experience.

Item	Dimension	Acceptance Factors	R, Item/Total Score Correlation	Alignment with PedBotHome Experience
1	Fit to Use	Adaptability to the spaces where one spends one’s everyday life (home, work)	0.724	Aligned
2	Fit to Use	Contribution to the improvement to one’s everyday life	0.695	Aligned (dual interpretation)
3	Ease of Use	Ease of learning all individual functions	0.354	Aligned (ambiguity noted)
4	Ease of Use	Ease of learning the basic functions (the functions that concern the individual more)	0.518	Aligned (ambiguity noted)
5	Ease of Use	Ease of use (complexity, required effort)	0.485	Aligned
6	Fit to Use	Security	0.681	Ambiguous
7	Fit to Use	Dimensions (height, width, length)	0.633	Aligned
8	Fit to Use	Weight	0.614	Aligned
9	Fit to Use	Sufficiency of functionality	0.465	Aligned
10	Fit to Use	Feeling protected, secure, confident	0.600	Ambiguous
11	Ease of Use	Feeling more autonomous	0.628	Aligned
12	Ease of Use	Needing help from another person to use	0.612	Aligned
13	Fit to Use	I will feel comfortable to use the assistive device around the community.	0.655	Not aligned
14	Ease of Use	Feeling comfortable using around colleagues (working environment)	0.732	Not aligned
15	Fit to Use	Feeling comfortable using around friends and family	0.719	Aligned
IF1	Individual Functionalities	Ease of use	0.946	Aligned
IF2	Individual Functionalities	Help provided in everyday life	0.991	Aligned (dual interpretation)
IF3	Individual Functionalities	Safety, security	0.993	Ambiguous
IF4	Individual Functionalities	Reliability	0.991	Aligned
IF5	Individual Functionalities	Feeling of safety	0.996	Ambiguous

**Table 5.** Exergame Engagement Factors Alignment with PedBotHome Experience and PYTHEIA [31] Scale Items.

Exergame Engagement Factors	Studies Reporting	Alignment with PedBotHome Experience	Alignment (Tentative) with PYTHEIA Scale Items
Overall Enjoyment			
Overall degree of game enjoyment/fun	Whittinghill and Brown, 2014; Radtka et al., 2013; Freitas et al., 2013	Aligned	Aligned. Item 2
Difficulty/ease of playing	Radtka et al., 2013; Freitas et al., 2013	Aligned	Aligned. Item 2
Physical Interface			
Range of motion and hold time diminish fun	Bryanton et al., 2006	Aligned	Aligned. IF1–5
Repetitions do not diminish fun	Bryanton et al., 2006	Ambiguous	Aligned. Item 2
Game controls are most difficult to get positively appraised	Whittinghill and Brown, 2014	Aligned	Aligned. Items IF1–5
Being comfortable while playing	Radtka et al., 2013	Aligned	Ambiguous. Potential partial alignment with Items 10 and 2.
Game and Scenario Graphics			
Visual aesthetic	Whittinghill and Brown, 2014	Aligned	Ambiguous. Potential alignment with items 2/ IF2.
Immersion in game	Radtka et al., 2013	Aligned	Ambiguous. Potential alignment with items 2/ IF-2.
Realism of look and feel of game	Radtka et al., 2013	Aligned	Not aligned
Enjoyment of game scenario	Freitas et al., 2013	Aligned	Ambiguous. Potential alignment with items 2/IF-2.
Overall Competence and Control			
Sense of competence playing game	Radtka et al., 2013	Aligned	Ambiguous. Potential partial alignment with items 3, 4, and 11.
Sense of control of game	Radtka et al., 2013; Freitas et al., 2012	Aligned	Ambiguous. Possible alignment with item 12.
Challenge of the game	Freitas et al., 2013	Aligned	Not aligned
Incentive From Therapeutic Awareness			
Perceived therapeutic function	Freitas et al., 2013	Aligned	Not aligned
Help game provided correctly doing therapeutic movements	Freitas et al., 2013	Not aligned	Not aligned
Game increased motivation to perform exercises	Freitas et al., 2013; Sandlund et al., 2011	Aligned	Not aligned
Spurred child’s initiative to exercise	Sandlund et al., 2011	Not aligned	Ambiguous. Potential partial alignment with item 11.

**Table 6.** PYTHEIA [31] scale individual functionality items applied to controller and airplane game in PedBotHome.

		Selected Functionality	
Item	Individual Functionality Rating Criteria with Respect to the Specified Functionality	Robotic Footplate Interface for Game Control (Hardware)	The Airplane Game (Software)
IF1	Rate your satisfaction in relation to its ease of use	Aligned	Aligned
IF2	Rate your satisfaction in relation to the help it provides in your everyday life.	Dual (Multiple) Alignments “Everyday Life” Interpretations (1) As supports functional improvement, i.e., exercises your ankle as it should (2) As works well for gameplay	Dual (Multiple) Alignments “Everyday Life” Interpretations (1) As is generally entertaining (2) As helps engagement in HEP
IF3	Rate your satisfaction in relation to how safe/secure it is.	Ambiguous–need to distinguish this risk from that of injury on the system (IF5) and unreliability of function (IF4).	Not aligned
IF4	Rate your satisfaction in relation to its reliability	Aligned Hardware reliability	Aligned Software reliability
IF5	Rate your satisfaction in relation to the feeling of safety it provides	Aligned Freedom from non-injurious malfunction	Not Aligned
