# Supplementary material for: Toward Evaluation of the Subjective Experience of a General Class of User-Controlled, Robot-Mediated Rehabilitation Technologies for Children with Neuromotor Disability
Source: Informatics (MDPI). Author manuscript; Available in PMC 2021 Sep 13. (PMC8436173; doi:10.3390/informatics7040045)
Supplement: Codebook - Adherence Theory - 8-31-2020 [file NIHMS1719237-supplement-Codebook_-_Adherence_Theory_-_8-31-2020.docx]

Adherence Theory

Nodes

| Name | Description |
| --- | --- |
| By Theory |  |
| Adherence Theory | Adherence theory to home exercise programs by families of children with CP Lillo-Navarro et al. 2015 |
| Exercise Factors |  |
| Exercise Amount |  |
| Family Burden |  |
| Family Disruption |  |
| Time |  |
| Exercise Preferences |  |
| Fun |  |
| Get Results |  |
| Pain-free |  |
| Simple to Do |  |
| Therapist Factors |  |
| Build Parent Confidence |  |
| Demonstrating Exercise |  |
| Give Info & Support |  |
| Providing Feedback |  |
| Written Instructions |  |
| Incentivizing Adherence |  |
| Achievement Perception |  |
| Change in Child's Exercise Performance |  |
| Incentive Based on Goals |  |
| Peace of Mind |  |
| Monitoring & Encouragement |  |
| Perception of Regular Monitoring |  |
| Promote Daily Routine | The "therapist" promotes incorporation of exercise into the daily routine |
| Reminders |  |
| Exergaming Engagement Theory |  |
| 27. ROM, Hold & Fun | Range of motion and hold time, but not repetitions, sensitive to perceived fun and enjoyment |
| 28. Visual aesthetic |  |
| 29, 39. Control | Game controls - most difficult to get a positive appraisal relative to. Relates to ZPD (Vygotsky) and to Flow State. Challenge balance. |
| 40. Therapeutic connection | Do you preceive the therapeutic function of the game? |
| 41. Correct Execution of Exercise | Do you preceive the therapeutic function of the game? |
| 45, 49, 69. Motivation to Exercise | Would the game improve your motivaton to perform exercises? |
| 48. Compliance |  |
| 46. Game scenario (story) | Did you enjoy the game scenario? |
| 47. Challenge | Did you feel challenged? |
| 50. Initiative |  |
| ZPD Criteria | Vgotsky |
| 31, 44, 50. Enjoyment, global | Not analyzed into components, global - How much did you enjoy playing the game? Do you think the game was fun? Global engagement. |
| 32. Immersion | How much did you feel you were a part of the game? |
| 33. Competence | Did you feel you were good at the game? |
| 34. Autonomy | How much did you feel in charge of what you were doing? |
| 35. Vraisemblance | Did the game look and feel real? |
| 36. Learnability | Did you get enough information from the game to play? Suggestion of "basic functions" link. |
| 37, 42. Comfort | Were you comfortable while you were playing? Did you feel comfortable during the playing experience? |
| 38, 43. Complexity and Effort | How hard was it to play the game? Did you find that the game was easy to play? |
| Subjective Assessment ART |  |
| 1. Adaptability |  |
| 10. Feelings of Security, Being Protected, Confident |  |
| 11. Autonomy |  |
| 12. Needing help from another person |  |
| 13. Comfortable using in community |  |
| 14. Comfortable using at work |  |
| 15. Comfortable using around friends, family |  |
| 2. Improvement to Daily Life |  |
| 3. Learning Individual (All) Functions |  |
| 4. Learning Basic Functions |  |
| 5. Ease of Use, Complexity, Effort |  |
| 6. Security |  |
| 7. Dimensions |  |
| 8. Weight |  |
| 9. Sufficiency of functionalities |  |
| IF1 Ease of use |  |
| IF2 Helpfulness in everyday life |  |
| IF3. Safety and Security |  |
| IF4. Reliability |  |
| IF5. Feeling of Security, Being Protected, Confident |  |
| Themes |  |
| Environmental Factors |  |
| Everyday Life Impact |  |
| Adaptability to Everyday Spaces | Home, work |
| Help in Everyday Life |  |
| Improvement to Everyday Life |  |
| Human-Social Support |  |
| Non-specific |  |
| Therapist |  |
| Building Parents' Confidence |  |
| Helping Incorporate into Daily Routine |  |
| Monitoring and Supporting Adherence |  |
| Therapist (Non-specific characteristics) |  |
| Intervention Program |  |
| Exercises-Movements |  |
| Amount of Exercise |  |
| Adherence - Compliance |  |
| Burden |  |
| Child Burden |  |
| Duration (General) |  |
| Family Burden (Time Intrusion) |  |
| Parental Burden |  |
| Performance |  |
| Changes in Performance | Positively, improvement in performance of exercise if not function |
| Correct Performance |  |
| Motivation to Perform |  |
| Association with Positive Results |  |
| Tasks (Specific Movements) |  |
| Hold Time |  |
| Nature of Exercises (General) |  |
| Reps |  |
| ROM Exercises |  |
| Tracking |  |
| Therapeutic Function |  |
| Target Technology |  |
| Discrete Features (Game) |  |
| Game Scenario |  |
| Immersion |  |
| Look and Feel |  |
| Discrete Features (Non Game) |  |
| Confidence Promotion |  |
| Ease of Use |  |
| Help in Everyday Life |  |
| Reliability |  |
| Safety and Security |  |
| Sufficiency of Functionality (Global) |  |
| Tech (Global) Dynamic Characteristics | In use, in performing function |
| Ease of Use |  |
| Complexity, Required Effort |  |
| Learnability |  |
| Basic (Most Desired) Functions |  |
| Individual Functions (All) |  |
| Reliability |  |
| Safety and Security |  |
| Tech (Global) Physical Characteristics |  |
| Dimensions |  |
| Weight |  |
| Personal Factors |  |
| Afective Response |  |
| Engagement |  |
| Enjoyment |  |
| Frustration |  |
| Fun |  |
| Happiness |  |
| Interest |  |
| Like - Dislike |  |
| Sense of Achievement |  |
| Sense of Confidence, Security | Being protected |
| Stimulation |  |
| Autonomy |  |
| Challenge |  |
| Competence |  |
| Control |  |
| Control - Of System Itself |  |
| Help from Another Person to Use |  |
| Internal Motivation | Alignment with goals, personality note motivation is also associated with the program itself |
| Personal Effort | As a personal characteristic mediating system use |
| Physical Response |  |
| Comfort |  |
| Fatigue |  |
| Feels Bad |  |
| Underlying Health |  |
| Self-conscious Use |  |
| Around Co-Workers |  |
| Around Family |  |
| Around Friends |  |
| In Community |  |
| Time Management |  |
| ZPD - Zone of Proximal Development | Analog to FLOW challenge moderation |
