# Supplementary material for: Toward Evaluation of the Subjective Experience of a General Class of User-Controlled, Robot-Mediated Rehabilitation Technologies for Children with Neuromotor Disability
Source: Informatics (MDPI). Author manuscript; Available in PMC 2021 Sep 13. (PMC8436173; doi:10.3390/informatics7040045)
Supplement: PYTHEIA Items and Scale Formats [file NIHMS1719237-supplement-PYTHEIA_Items_and_Scale_Formats.docx]

Table 1. PYTHEIA Items

| Item # | Item Text *(As translated from the Greek in Koumpouros et al. 2016)* |
| --- | --- |
| IF1 | Rate your satisfaction with the specific feature of your assistive device in relation the ease of use. |
| IF2 | Rate your satisfaction with the specific feature of your assistive device in relation to the help it provides in your everyday life. |
| IF3 | Rate your satisfaction with the specific feature of your assistive device in relation to how safe/secure it is. |
| IF4 | Rate your satisfaction with the specific feature of your assistive device in relation to its reliability (i.e. whether it applies always correctly). |
| IF5 | Rate your satisfaction with the specific feature of your assistive device in relation to the feeling of safety (I will feel more secure, protected, confident when using it). |
| 1 | Rate your satisfaction with the supporting device and the services provided in relation to the adaptability in the spaces you spend your everyday life (home, work). |
| 2 | Rate your satisfaction with the supporting device and the services provided in relation to its contribution to the improvement of your everyday life. |
| 3 | Rate your satisfaction with the supporting device and the services provided in relation to the ease of learning all individual functions. |
| 4 | Rate your satisfaction with the supporting device and the services provided in relation to the ease of learning the basic functions (the functions that concern me more). |
| 5 | Rate your satisfaction with the supporting device and the services provided in relation to the ease of use (complexity, required effort). |
| 6 | Rate your satisfaction with the supporting device and the services provided in relation to how secure it is. |
| 7 | Rate your satisfaction with the supporting device and the services provided in relation to the dimensions (height, width, length). |
| 8 | Rate your satisfaction with the supporting device and the services provided in relation to the weight. |
| 9 | Rate your satisfaction with the supporting device and the services provided in relation to if the functionalities existing are sufficient. |
| 10 | I will feel more secure (protected, confident) when using this assistive device. |
| 11 | I will feel more autonomous when using this assistive device. |
| 12 | I will need help from another person to use the assistive device. |
| 13 | I will feel comfortable to use the assistive device around the community. |
| 14 | I will feel comfortable to use the assistive device among my colleagues (working environment). |
| 15 | I will feel comfortable to use the device around friends and family. |

Table 2. PYTHEIA Item Scale Format

| ***Items*** | ***0*** | ***1*** | ***2*** | ***3*** | ***4*** | ***5*** |
| --- | --- | --- | --- | --- | --- | --- |
| ***IF1-IF5*** | *N/A* | *Not at all satisfied* | *Slightly satisfied* | *Moderately satisfied* | *Very Satisfied* | *Extremely satisfied* |
| ***1-9*** | *N/A* | *Not at all satisfied* | *Slightly satisfied* | *Moderately satisfied* | *Very Satisfied* | *Extremely satisfied* |
| ***10-15*** | *N/A* | *Not at all (0% of the time)* | *Sometimes (around 25% of the time)* | *Half the time, neutral (about 50% of the time)* | *Often (around 75% of the time)* | *All the time (100% of the time)* |
